# Discrete Isothermic Nets Based on Checkerboard Patterns

**DOI:** 10.1007/s00454-023-00558-1

**Published:** 2023-09-14

**Authors:** Felix Dellinger

**Affiliations:** 1 Institute of Geometry, TU Graz, Kopernikusgasse 24, 8010 Graz, Austria; 2 Institute of Geometry and Discrete Mathematics, TU Wien, Wiedner Hauptstraße 8-19/104, 1040 Vienna, Austria

**Keywords:** Differential geometry, Isothermic surfaces, Discrete differential geometry, Koenigs nets, 51B10, 53A99

## Abstract

This paper studies the discrete differential geometry of the checkerboard pattern inscribed in a quadrilateral net by connecting edge midpoints. It turns out to be a versatile tool which allows us to consistently define principal nets, Koenigs nets and eventually isothermic nets as a combination of both. Principal nets are based on the notions of orthogonality and conjugacy and can be identified with sphere congruences that are entities of Möbius geometry. Discrete Koenigs nets are defined via the existence of the so-called conic of Koenigs. We find several interesting properties of Koenigs nets, including their being dualizable and having equal Laplace invariants. Isothermic nets can be defined as Koenigs nets that are also principal nets. We prove that the class of isothermic nets is invariant under both dualization and Möbius transformations. Among other things, this allows a natural construction of discrete minimal surfaces and their Goursat transformations.

## Introduction

Discretizing principal curvature nets is of great interest not only from a differential geometric point of view, but also in geometry processing, computer graphics and even freeform architecture [14, 18]. The most prominent versions of discrete principal nets are circular nets and conical nets [4, 14]. A new discretization was introduced in [2] and later in [17], independently. In [2] principal nets are defined as a pair of planar quadrilateral nets with orthogonal corresponding edges. This approach can be extended to *n*-dimensional space and was driven by the discrete Euler–Poisson–Darboux equation. The authors could show that their discrete confocal coordinates are the separable orthogonal solutions of the discrete Euler–Poisson–Darboux equation in analogy to the smooth theory. Coming from a completely different angle, the discretization of principal curvature nets in [17] is based on the checkerboard pattern inscribed in a quadrilateral net constructed by connecting edge midpoints. This approach has already proven to be useful in various applications [10, 11, 16]. Its effectiveness suggests that there is more to the concept than just the good numerical approximation qualities already hinted at in [17]. Indeed, checkerboard patterns are equivalent to pairs of classical nets and as such have been used in [2, 3], providing equivalent definitions of principal nets. This paper contributes further to the discrete theory introduced in [2, 3] while adopting the point of view of [17].

A checkerboard pattern is a quadrilateral net where every second face is a parallelogram. The edges of these parallelograms can be seen as discrete derivatives. If all faces in between the parallelograms are planar we speak of a conjugate checkerboard pattern. If additionally all parallelograms are rectangles we speak of a principal checkerboard pattern. As the concept of checkerboard patterns is Euclidean in nature, it is surprising that principal nets are Möbius invariant if they are seen as sphere congruences [19]. Lifting these sphere congruences to the projective model of Möbius geometry preserves principality and offers the appropriate environment to efficiently study these geometric objects.

For a net with planar faces the supporting lines of neighboring edges intersect. Every face can be associated with six such intersection points. In [7] discrete Koenigs nets have been characterized by the property that these six points lie on a common conic section, the so-called conic of Koenigs [12]. We apply this definition to a checkerboard pattern. The resulting discrete Koenigs nets enjoy several interesting properties such as projective invariance and the existence of dual nets similar to the approach in [5]. Usually, Koenigs nets have been known as *nets with equal Laplace invariants*. While this property has been lost with previous discretizations of Koenigs nets, we manage to retain it in a natural way.

We define discrete isothermic nets as discrete Koenigs nets that are also principal. Analogous to the classical smooth theory, the class of discrete isothermic nets is invariant under both dualization and Möbius transformations. This is not only interesting from a theoretical point of view, but also offers a practical way to define and construct discrete minimal surfaces as surfaces that are dual to their own Gauß image. Consequently, the dual of any isothermic net on the unit sphere can be seen as a minimal surface. All of these steps can now be easily discretized with our approach.

## Checkerboard Patterns

### Preliminaries

**Fig. 1:**
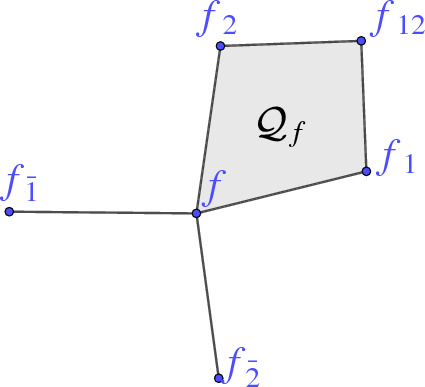
Notation for vertices and faces

In this paper we study two-dimensional nets $f :D \rightarrow {\mathbb {R}}^3$. All our constructions are local which is why we can always assume $D={\mathbb {Z}}^2$. To denote the one-ring or two-ring neighborhood of a vertex *f*(*k*, *l*) we use the *shift notation* as can be seen in Fig. 1. The index *i* resp. $\bar{i}$ indicates that the *i*-th coordinate is increased resp. decreased by one with $i \in \{1,2\}$. For instance, 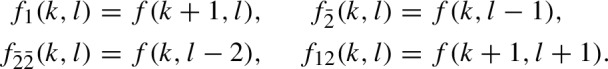 We call the images of *f* the vertices and the pairs $(f,f_1)$ or $(f,f_2)$ the edges of the net. Further we denote by $\mathcal {Q}_f$ the face $(f, f_1, f_{12}, f_2)$. If no confusion can arise, we drop the index and just write $\mathcal {Q}$.

#### Definition 2.1

A *checkerboard pattern* is a regular quad net where every second face is a parallelogram: $\mathcal {Q}_{f}(k,l)$ is a parallelogram if $k+l\equiv 0$ (mod 2).

**Fig. 2:**
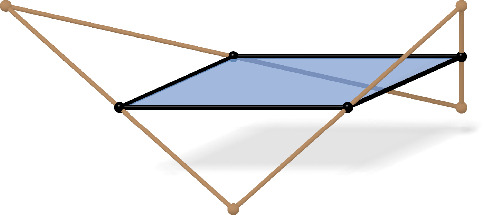
An inscribed first order face, which is always a parallelogram

Even if at first glance the definition of checkerboard patterns seems quite restrictive, they are actually very natural objects. From any given net $f$ we can easily construct a checkerboard pattern $c_{f}$ by midpoint subdivision as described in [17]: The vertices of $c_{f}$ are the edge midpoints of $f$. There are then two kinds of faces in $c_{f}$. The first type of face is formed by the midpoints of edges of each face $\mathcal {Q}$ of $f$ (compare Fig. 2). It is elementary that these faces are parallelograms whose edges are parallel to the two diagonals of $\mathcal {Q}$. We will refer to them as first order faces, as their edges can be interpreted as discrete first order derivatives. We denote the first order face associated to the quadrilateral $\mathcal {Q}_f(k,l)$ by $\mathcal {B}_{f}(k,l)$.

**Fig. 3:**
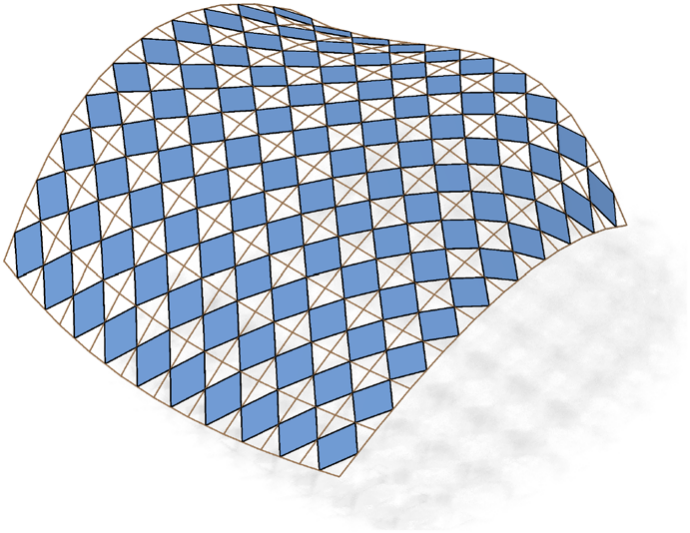
The first order faces of the checkerboard pattern are the blue parallelograms $\mathcal {B}_{f}$ inscribed in the faces of the control net $f$. The white quadrilaterals $\mathcal {W}_{f}$ in between are the second order faces

The second type of face is formed by the midpoints of edges emanating from a common vertex of $f$. Those faces are, in general, non-planar quadrilaterals. We will refer to them as second order faces, because we associate properties related to second order derivatives with them. The second order face associated to the vertex *f*(*k*, *l*) will be denoted by $\mathcal {W}_{f}(k,l)$, compare Fig. 3.

**Fig. 4:**
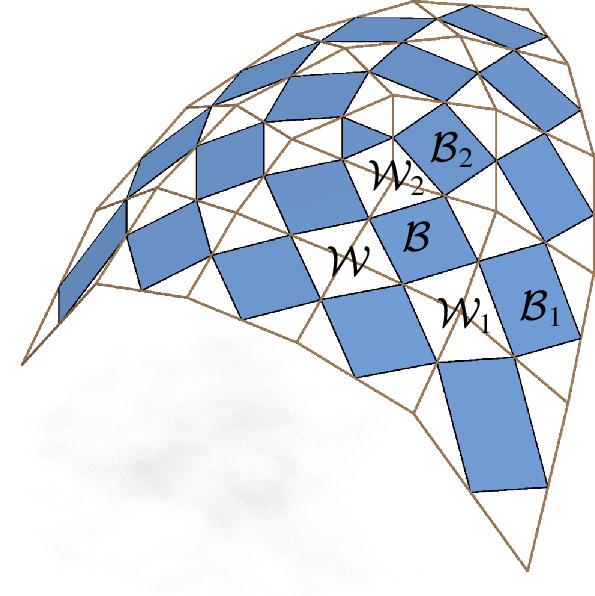
Control net and associated checkerboard pattern with a combinatorial singularity

If no confusion can arise we will drop the index $f$ in all quantities. Following [17], we call $c_{f}$ the checkerboard pattern of $f$ and $f$ the control net of $c_{f}$, see Fig. 4.

#### Remark 2.2

**Fig. 5:**
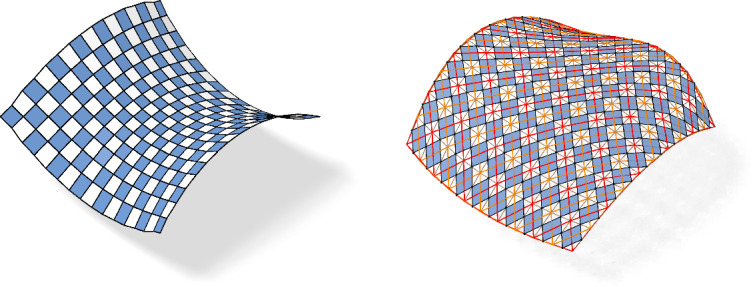
Left: A principal checkerboard pattern. All the white faces are planar and all blue faces are rectangles. Right: The two nets defined by the diagonals of the control net have planar faces if and only if the checkerboard pattern is conjugate

For a given checkerboard pattern there is a three-parameter family of control nets. A control net is uniquely determined after the choice of an initial vertex as all other vertices can be obtained through iterated reflection at the vertices of the checkerboard pattern. However, the two nets defined via the diagonals of the control net are always defined uniquely by the checkerboard pattern up to translation, compare Fig. 5, right. Consequently any property of the checkerboard pattern can be traced back to the diagonal nets and vice versa. Building the theory from the point of view of the diagonal nets is the approach chosen in [2, 3]. This seems more suitable for higher dimensional nets but as the focus of this paper is on nets in three-dimensional space the author sticks to checkerboard patterns due to their intuitive visualization.

#### Remark 2.3

The checkerboard pattern approach can be extended to nets with combinatorial singularities. For each *n*-gon, midpoint subdivision creates an inscribed *n*-gon, see e.g. an inscribed triangle in Fig. 4.

For $\epsilon >0$, let the net $f:{\mathbb {Z}}^2 \rightarrow {\mathbb {R}}^3$ sample a smooth surface parametrization $\phi :{\mathbb {R}}^2\rightarrow {\mathbb {R}}^3$, i.e., $f(k,l)=\phi (\epsilon k,\epsilon l)$. We define the directionsIntuitively speaking, the parameter lines of *f* and $c_{f}$ enclose an angle of 45 degrees. So, we can think of $c_{f}$ as being parameterized along the directions *u* and *v* in the coordinate plane. The *edge vectors*of $\mathcal {B}_{f}(k,l)$ approximate the directional derivatives $\partial _u \phi$ and $\partial _v\phi$ at , up to second order. Indeed,as a simple Taylor expansion shows. Moreover, it can be shown by Taylor expansion that the difference of opposite edge vectors in a second order face $\mathcal {W}_{f}(k,l)$ approximates $\partial _{uv}\phi (\epsilon k,\epsilon l)$ by first order. This motivates the notation of $\delta _u f$ and $\delta _v f$ for the edge vectors of $\mathcal {B}_{f}$ and gives rise to the following definition.

#### Definition 2.4

We call a checkerboard pattern *orthogonal* if its first order faces are rectangles. We call it *conjugate* if its second order faces are planar. A checkerboard pattern is *principal* if it is both conjugate and orthogonal, compare Fig. 5, left.

#### Remark 2.5

Conjugacy of a checkerboard pattern $c_{f}$ is already determined by its control net $f$ and so are orthogonality and principality. Indeed, second order faces of $c_{f}$ are planar if and only if the two nets defined by the diagonals of *f* have planar faces, compare Fig. 5, right. Thus the class of conjugate checkerboard patterns is invariant under projective transformations applied to the vertices of the control net.

## Curvature Theory

**Fig. 6:**
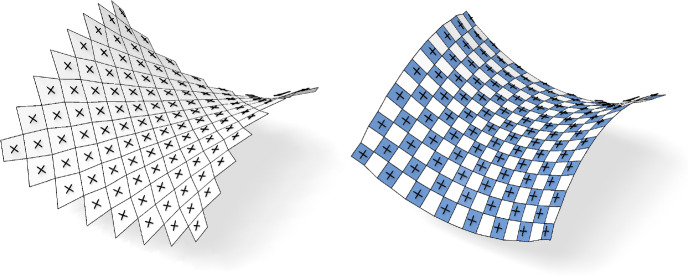
Left: A control net of a principal checkerboard pattern and the eigenvectors of the shape operator. Right: The checkerboard pattern of the same net. We see that the first order faces are aligned with the eigenvectors of the shape operator as stated by Corollary 3.5

**Fig. 7:**
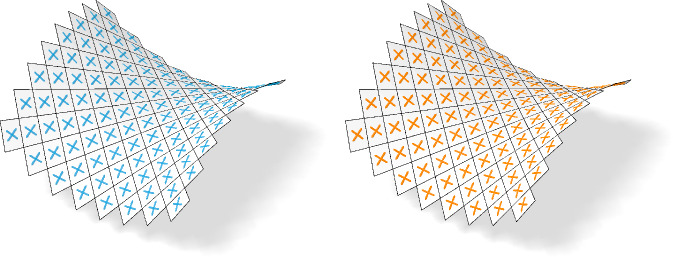
If the net $f$ samples a smooth parametric surface $\phi$, the underlying checkerboard pattern can be used to compute the discrete principal curvature directions (left) which are visually not distinguishable from the analytically computed directions (right)

In this section, we define a discrete version of the shape operator connecting nets to their Gauß images. We find that the properties of the shape operator for conjugate or principal nets are consistent with the smooth theory, see Fig. 6. Moreover, the discrete shape operator provides a way to numerically approximate smooth principal curvature directions, compare Fig. 7. We start by defining the Gauß image of a net.

### Definition 3.1

Let $f$ be a net. Then

$$\begin{aligned} n = \frac{(f_{1} - f_{\bar{1}})\times (f_2 - f_{\bar{2}})}{\Vert (f_{1} - f_{\bar{1}})\times (f_2 - f_{\bar{2}})\Vert } \end{aligned}$$

is a net with vertices on the unit sphere ${\mathbb {S}}^2$. We call *n* the *Gauß image* or *vertex normals* of $f$. Additionally, for the face $\mathcal {Q}_f = (f,f_1,f_{12},f_2)$ we define the *face normal* *N* by1The *generalized surface area* of $\mathcal {Q}_f$ is the surface area of the orthogonal projection of $\mathcal {Q}_f$ in direction of *N*,2

### Remark 3.2

For planar quadrilaterals without self-intersections the generalized surface area is the same as the surface area. The face normal *N* is a normal vector to $\mathcal {B}_{f}$ and for a planar face $\mathcal {Q}$ it coincides with a normal vector to $\mathcal {Q}$. The vertex normal *n* at $f$ is also the face normal of the corresponding second order face $\mathcal {W}_{f}$ in the sense of formula (1).

Having defined a Gauß image *n* for a net $f$, we can relate the discrete derivatives $(\delta _u f,\delta _v f)$ and $(\delta _u n,\delta _v n)$ with the help of the corresponding checkerboard patterns $c_{f}$ and $c_{n}$. The idea is to define the shape operator as the linear mapping $(\delta _u f,\delta _v f)\mapsto (\delta _u n,\delta _v n)$. However, we face the problem that $(\delta _u f,\delta _v f)$ and $(\delta _u n,\delta _v n)$ not necessarily span the same two-dimensional subspace. This is overcome by projecting in the direction of *N*, leading to the following definition:

### Definition 3.3

Let $f$ be a net, let $n_{f}$ be its Gauß image and let $P_N$ be the orthogonal projection along the corresponding face normal *N*. We define $S$ as the function on ${\mathbb {Z}}^2$ that maps (*k*, *l*) to a linear operator in the space spanned by $(\delta _u f,\delta _v f)$ such that

$$\begin{aligned} S(\delta _u f,\delta _v f) = P_N (\delta _u n,\delta _v n), \end{aligned}$$

where all entities are evaluated at a point $(k,l)\in {\mathbb {Z}}^2$. We call $S(k,l)$ the *shape operator* of the face $\mathcal {Q}_{f}(k,l)$. If no confusion can arise we drop the argument (*k*, *l*). The eigenvalues of $S(k,l)$ are denoted by the symbols $\kappa _1$ and $\kappa _2$ and are called the *principal curvatures*. The eigenvectors of $S(k,l)$ are the *principal curvature directions*.

**Fig. 8:**
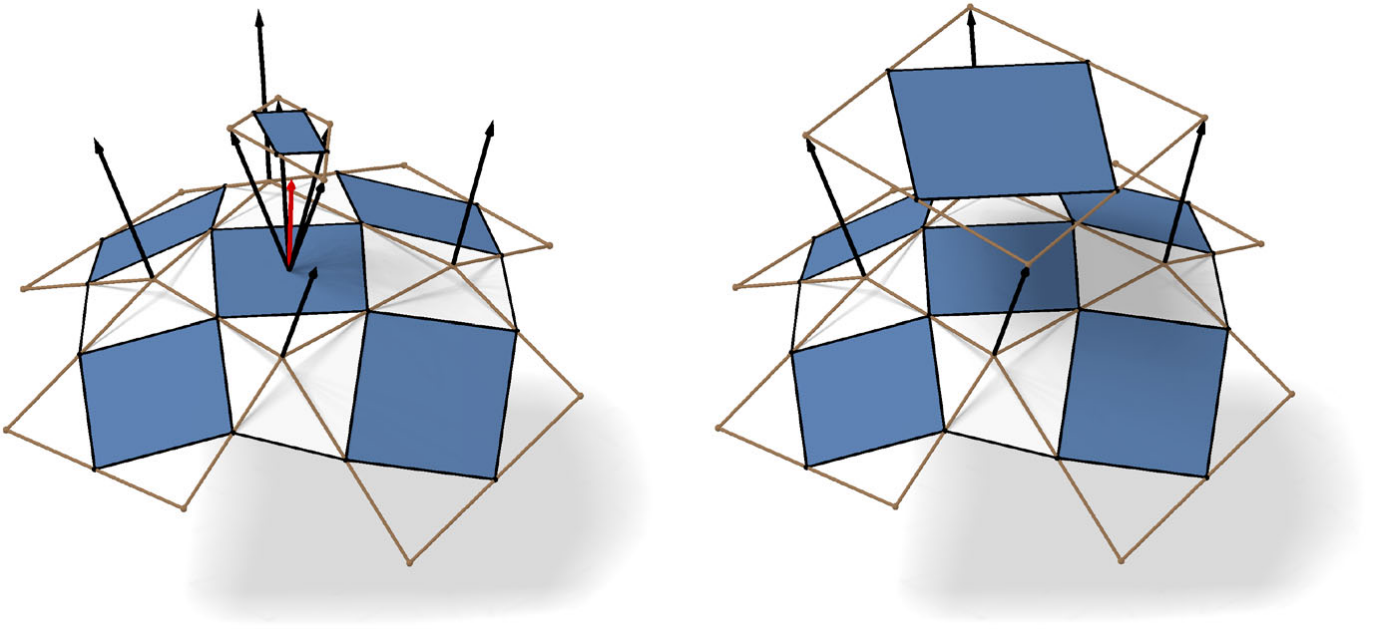
Left: A face $\mathcal {Q}_f$ of *f* and the corresponding Gauß image $\mathcal {Q}_n$. The shape operator maps the first order face $\mathcal {B}_{f}$ to the first order face $\mathcal {B}_{n}$ projected into the plane of $\mathcal {B}_{f}$. Right: The face $\mathcal {Q}$ and its offset $\mathcal {Q}^t$

For each face $\mathcal {Q}$ we can define an offset face $\mathcal {Q}^t$ by intersecting the plane parallel to $\mathcal {B}_{f}$ at distance *t* with the lines spanned by the vertices of $\mathcal {Q}$ and their corresponding vertex normals *n*, compare Fig. 8, left. Similarly to [9, 15, 18], the area of $\mathcal {Q}^t$ can be expressed by the *Steiner formula*3which can be shown by short algebraic manipulations.

### Lemma 3.4

For a conjugate checkerboard pattern the identities $\langle S\delta _{u} f , \delta _{v} f \rangle = \langle \delta _u f , S\delta _{v} f \rangle = 0$ hold. Thus the shape operator is symmetric.

### Proof

For a conjugate checkerboard pattern $c_{f}$ the Gauß image *n* is the normal vector of the corresponding second order face $\mathcal {W}_{f}$. Thus, it is orthogonal to all the edges that $\mathcal {W}_{f}$ shares with neighboring first order faces. As $\mathcal {B}_{f}$ is a parallelogram, both $n_f$ and $(n_f)_{12}$ are orthogonal to the edge $\delta _v f$. We find thatThe same argument applies to $\langle S\delta _v f , \delta _u f \rangle$. As $\delta _{u} f,\delta _{v} f$ constitute a basis of the domain of the shape operator, the shape operator is symmetric. $\square$

### Corollary 3.5

For a principal checkerboard pattern the edge vectors $(\delta _u f,\delta _v f)$ of $\mathcal {B}_{f}$ are eigenvectors of the shape operator.

### Proof

This follows immediately from $\langle S\delta _{u} f , \delta _{v} f \rangle = 0 = \langle \delta _{u} f , \delta _{v} f \rangle$. $\square$

As the partial derivatives can be observed in first order faces, so can the first fundamental form ${\text {I}}$. By using the first order face  of the Gauß image and the corresponding derivatives $\delta _u n$ and $\delta _v n$ we can analogously define a second fundamental form.

### Definition 3.6

Consider a net $f$ and its Gauß image *n*. We define the *first* and *second fundamental forms* by letting

$$\begin{aligned} {\text {I}}:= \begin{pmatrix} \langle \delta _u f , \delta _u f \rangle &{} \langle \delta _u f , \delta _v f \rangle \\ \langle \delta _v f , \delta _u f \rangle &{} \langle \delta _v f , \delta _v f \rangle \end{pmatrix}, \quad \ {\text {II}}:= \begin{pmatrix} \langle \delta _u f , \delta _u n \rangle &{} \langle \delta _u f , \delta _v n \rangle \\ \langle \delta _v f , \delta _u n \rangle &{} \langle \delta _v f , \delta _v n \rangle \end{pmatrix}. \end{aligned}$$

### Lemma 3.7

A matrix representation $\Sigma$ of the shape operator with respect to the basis $(\delta _u f, \delta _v f)$ is given by $\Sigma ={\text {I}}^{-1} {\text {II}}$.

### Proof

When using coordinates with respect to $(\delta _u f,\delta _v f)$ the inner product $\langle \,{\cdot }\, , \,{\cdot }\, \rangle$ is represented by the coordinate matrix ${\text {I}}$. For any vector  we have $\langle v , \delta _u n \rangle = \langle v , P_N \delta _u n \rangle$ and likewise for $\delta _v n$. Thus the bilinear form $\langle \,{\cdot }\, , S\,{\cdot }\, \rangle$ is represented by the coordinate matrix ${\text {II}}$. For two vectors $\textbf{w}_1$ and $\textbf{w}_2$ with coordinates $w_1$ and $w_2$ we find that

$$\begin{aligned} w_1^T {\text {II}}w_2 = \langle \textbf{w}_1 , S \textbf{w}_2 \rangle = w_1^T {\text {I}}\Sigma w_2. \end{aligned}$$

It follows that ${\text {II}}= {\text {I}}\Sigma$. $\square$

### Remark 3.8

Due to Lemma 3.4, in a conjugate checkerboard pattern the second fundamental form is a diagonal matrix.

In analogy to [15] and [9] the area defined in Definition 3.1 can be computed by a mixed area form. This motivates the following definition.

### Lemma and Definition 3.9

Let $A(\,{\cdot },\,{\cdot }\,)$ be the *mixed area form* defined by4for two quadrilaterals with the same normal $N_f = N_g$. Then  holds.

The mixed area form is closely related to the mean and Gaußian curvatures.

### Lemma 3.10

For a net $f$ and its Gauß image *n* we define $\widetilde{\mathcal {Q}}_n$ as the orthogonal projection of $\mathcal {Q}_n$ onto the supporting plane of $\mathcal {B}_{f}$. The following identities hold:

### Proof

These identities can be shown by algebraic manipulations, in particular making use of the Lagrange identity$\square$

### Remark 3.11

Definition 3.1 requires that every normal vector lies exactly on the unit sphere. For principal nets one can relax this requirement and instead adapt the lengths of normal vectors, such that the first order face  of $n$ is parallel to the first order face $\mathcal {B}_{f}$ of $f$, as we will see in Sect. 4.3. This does not change principal directions and the Steiner formula (3) still holds.

## Möbius Transformations of Checkerboard Patterns

This section discusses a way to apply Möbius transformations to orthogonal nets. This was originally introduced by [19], who showed that the orthogonality of a net is equivalent to the existence of a sphere congruence of orthogonally intersecting spheres. A Möbius transformation can then be applied to these spheres, and from the transformed congruence we can obtain the transformed orthogonal net. We show that the class of principal nets is invariant under such Möbius transformations. A fact that is also evident form the higher dimensional point of view developed in [19], but we present a proof adjusted to our setting. Moreover, the orthogonal sphere congruence allows us to embed principal nets in the projective model of Möbius geometry ${\mathbb {P}\mathbb {R}^{4,1}}$. This turns out to be a powerful tool for studying principal nets and gives rise to a non-Euclidean generalization of discrete principal nets.

We write $s = (c,r^2)$ for a sphere *s* with center $c\in {\mathbb {R}}^3$ and squared radius $r^2 \in {\mathbb {R}}$. Two spheres $s_1 = (c_1,r_1^2)$ and $s_2 = (c_2, r_2^2)$ intersect orthogonally, if and only if

5 $$\begin{aligned} \langle c_1-c_2 , c_1 -c_2 \rangle = r_1^2 + r_2^2. \end{aligned}$$

**Fig. 9:**
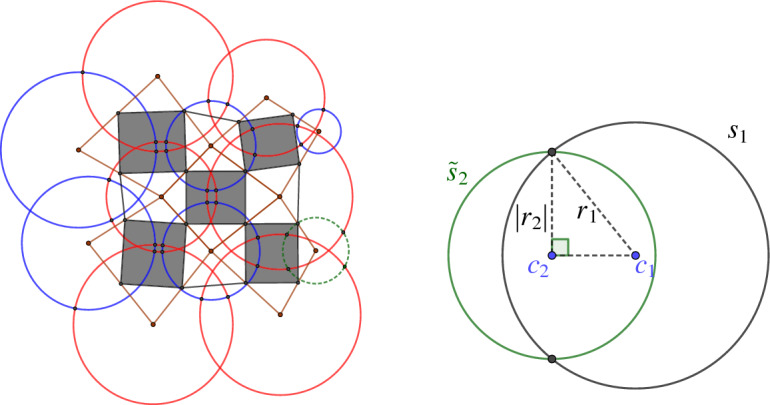
Left: The Möbius representation of a two-dimensional orthogonal net. Every red circle intersects every neighboring blue circle orthogonally and vice versa. The green dashed circle represents a circle with negative squared radius. Right: The real valued circle $s_1 = (c_1,r_1^2)$ intersects the imaginary circle $s_2 = (c_2,r_2^2)$ with $r_2^2<0$ orthogonally if and only if it intersects the real valued circle $\tilde{s}_2 = (c_2, -r_2^2)$ in antipodal points

Note that by definition this extends to spheres of negative squared radii. We can interpret this in the projective model of Möbius geometry by including the points inside the light cone as will be explained in more detail later in this section. A sphere $s_1=(c_1, r_1^2)$ with $r_1^2\geqslant 0$ intersects a sphere $s_2 = (c_2, r_2^2)$ with $r_2^2<0$ orthogonally if and only if $s_1$ intersects the sphere $(c_2, -r_2^2)$ in antipodal points. This allows a geometric interpretation of the spheres with negative squared radius. See Fig. 9 for a two-dimensional example. This setup allows for the following lemma and definition.

### Lemma and Definition 4.1

Let *f* be a net and $r^2:{\mathbb {Z}}^2 \rightarrow {\mathbb {R}}$. We call the function $s = (f,r^2)$ a sphere congruence and interpret it as a family of spheres with centers in *f* and *possibly imaginary* radius *r*. The checkerboard pattern of $c_{f}$ is orthogonal if and only if there exists a one-parameter family of sphere congruences $s=(f,r^2)$ such that neighboring spheres intersect orthogonally. We call such a sphere congruence the *Möbius representation* $s_{f}$ of $f$ and $c_{f}$. If the checkerboard pattern associated to a sphere congruence *s* is principal, we call *s* a *principal sphere congruence*.

### Proof

Consider a quadrilateral $\mathcal {Q}= (f, f_1, f_{12}, f_2)$. We fix the squared radius $r^2$ of $s=(f,r^2)$ at an initial point $(k,l) \in {\mathbb {Z}}^2$. This uniquely determines the radii $r_1$ and $r_2$ since $r_i^2 = \langle f - f_i , f - f_i \rangle - r^2$ for $i \in \{1,2\}$. Now, an easy computation shows that

$$\begin{aligned} \langle f_{12} - f_1 , f_{12} - f_1 \rangle - r_1^2&= \langle f_{12} - f_2 , f_{12} - f_2 \rangle - r_2^2 \\&\iff \quad \langle f - f_{12} , f_1 - f_2 \rangle = 0. \end{aligned}$$

Hence, the radius $r_{12}$ is well defined if and only if the checkerboard pattern $c_{f}$ is orthogonal. This process can be continued unambiguously, so every radius only depends on the choice of the initial radius. $\square$

### Remark 4.2

If the domain of the net $f$ is not simply connected, the orthogonal sphere congruences $s_{f}$ do not exist in general. It is an interesting question for further research which properties might guarantee the existence of a Möbius representation for more complex topology or for combinatorial singularities.

### Lemma and Definition 4.3

Let $f$ be the control net of an orthogonal checkerboard pattern $c_{f}$ and let $s_{f}$ be its Möbius representation. The image of $s_{f}$ under a Möbius transformation is again an orthogonal sphere congruence with a corresponding net $f'$ and checkerboard pattern $c_{f'}$. We call $f'$ resp. $c_{f'}$ a *Möbius transformation* of $f$ resp. $c_{f}$.

### Theorem 4.4

Principal checkerboard patterns are mapped to principal checkerboard patterns under Möbius transformations.

### Proof

This follows directly from Theorem 4.7 as will be explained later on in this section. $\square$

### Projective Model of Möbius Geometry

To prove Theorem 4.4 we embed the Möbius representation $s_{f}$ of a principal checkerboard pattern $c_{f}$ into the projective model of Möbius geometry. A concise introduction to Möbius geometry can be found in [4]. For an extensive presentation of the theory the reader is referred to [8]. We briefly state the key points for this paper.

Let $\textbf{e}_1,\dots , \textbf{e}_5$ be the canonical basis vectors of the five-dimensional Minkowski space ${\mathbb {R}}^{4,1}$. It is equipped with the inner product

$$\begin{aligned} \langle \!\langle \textbf{e}_i, \textbf{e}_j \rangle \!\rangle = {\left\{ \begin{array}{ll} 1 &{} i = j \leqslant 4, \\ 0 &{} i \ne j,\\ -1 &{} i = j = 5. \end{array}\right. } \end{aligned}$$

For $x \in {\mathbb {R}}^{4,1}\setminus \{0\}$ we write [*x*] for the one-dimensional subspace spanned by *x*, i.e.,

$$\begin{aligned} {[}x] = \{ y \in {\mathbb {R}}^{4,1}: y = \lambda x,\, \lambda \in {\mathbb {R}}\}. \end{aligned}$$

We write ${\mathbb {P}\mathbb {R}^{4,1}}$ for the space of these one-dimensional subspaces. Any sphere $s = (c,r^2)$ can be identified with a point of ${\mathbb {P}}{\mathbb {R}}^{4,1}$ by the mapping

$$\begin{aligned} \iota (s) = \biggl [\biggl (c, \frac{|c|^2 - r^2 - 1}{2}, \frac{|c|^2 - r^2 + 1}{2}\biggr ) \biggr ] \in {\mathbb {P}}{\mathbb {R}}^{4,1}. \end{aligned}$$

We can view *c* as a vector in ${\mathbb {R}}^{4,1}$ where the fourth and fifth components are zero. By defining the vectors $\textbf{e}_0:= (\textbf{e}_5 - \textbf{e}_4)/2$ and $\textbf{e}_{\infty }:= (\textbf{e}_4 + \textbf{e}_5)/2$, we can writePoints can be seen as spheres with radius zero, so $\iota$ extends to points in ${\mathbb {R}}^3$. Observe that $\langle \!\langle \iota (s),\iota (s) \rangle \!\rangle =r^2$. Thus the set of spheres with radius zero is identified with the light cone ${\mathcal {L}}:=\{x\in {\mathbb {P}\mathbb {R}^{4,1}}:\langle \!\langle x,x\rangle \!\rangle = 0\}$. The points inside the light cone are those with $\langle \!\langle x, x\rangle \!\rangle < 0$ and correspond to spheres with negative squared radii.

From a Möbius geometric point of view, planes in ${\mathbb {R}}^3$ are spheres with infinite radius and center at infinity. We write $\epsilon = (n,d)$ for the plane defined by the equation $\langle n, x \rangle = d$. The mapping $\iota$ can now be extended to spheres with infinite radius (i.e., planes) by

$$\begin{aligned} {\iota (\epsilon ) = [n + 0\hspace{1.38885pt}{\cdot }\hspace{1.38885pt}\textbf{e}_0 + 2d \textbf{e}_{\infty }].} \end{aligned}$$

The advantage of the projective model of Möbius geometry lies in the well-known linearization of orthogonal intersection and Möbius transformations [4].

#### Theorem 4.5

Two spheres $s_1$ and $s_2$ in ${\mathbb {R}}^3$ with squared radii in ${\mathbb {R}}\cup \{\infty \}$ intersect orthogonally if and only if $\langle \!\langle \iota (s_1),\iota (s_2)\rangle \!\rangle = 0$. If one sphere has radius 0, orthogonal intersection is equivalent to just intersection. Möbius transformations in ${\mathbb {R}}^3$ canonically extended to spheres and planes are exactly the orthogonal transformations in ${\mathbb {P}\mathbb {R}^{4,1}}$.

#### Definition 4.6

Let $g$ be a net ${\mathbb {Z}}^2 \rightarrow {\mathbb {P}}{\mathbb {R}}^{4,1}$. If adjacent vertices are orthogonal, i.e., $\langle \!\langle g, g_1\rangle \!\rangle = \langle \!\langle g, g_2\rangle \!\rangle = 0$, and the corresponding checkerboard pattern $c_{g}$ is conjugate, we call $g$ a *pseudo-principal net in* ${\mathbb {P}}{\mathbb {R}}^{4,1}$. In order to avoid confusion we will denote nets in ${\mathbb {P}}{\mathbb {R}}^{4,1}$ by $g$, while we use $f$ for nets in ${\mathbb {R}}^3$.

Let $f$ be the control net of an orthogonal checkerboard pattern and let $s_{f}$ be a corresponding sphere congruence. Then $\iota \circ s_{f}$ is a net ${\mathbb {Z}}^2 \rightarrow {\mathbb {P}\mathbb {R}^{4,1}}$, where the vertices are the images of $s_{f}$ under $\iota$.

#### Theorem 4.7

If $s_{f}$ is a principal sphere congruence in ${\mathbb {R}}^3$, then $\iota (s_{f})$ is a pseudo-principal net in ${\mathbb {P}}{\mathbb {R}}^{4,1}$. If $g$ is a pseudo-principal net in ${\mathbb {P}}{\mathbb {R}}^{4,1}$, then $\iota ^{-1}(g)$ is a principal sphere congruence in ${\mathbb {R}}^3$.

#### Proof

Orthogonality of adjacent vertices of a net in ${\mathbb {P}}{\mathbb {R}}^{4,1}$ is equivalent to the orthogonal intersection of adjacent spheres in ${\mathbb {R}}^3$.

**Fig. 10:**
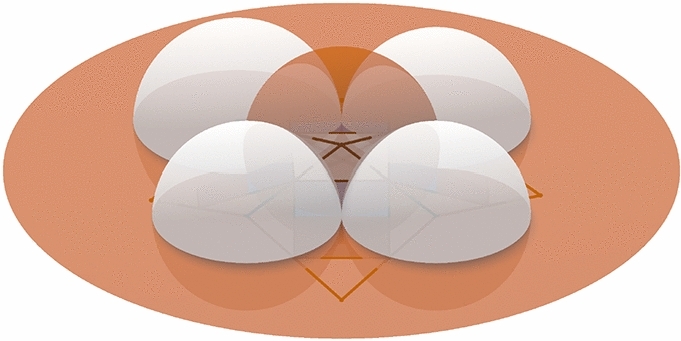
This figure illustrates why conjugacy of a checkerboard pattern is preserved under Möbius transformations. The four gray spheres are intersected orthogonally by the pencil spanned by the orange sphere and the orange plane. After applying a Möbius transformation the four gray spheres still intersect a pencil orthogonally which contains a plane. Hence the centers of the transformed spheres are still coplanar

Let $s_{f}$ be a principal sphere congruence in ${\mathbb {R}}^3$. The four spheres $s_{\bar{1}}$, $s_{\bar{2}}$, $s_1$, and $s_2$ all intersect both *s* and the plane $\epsilon$ spanned by the centers $f_{\bar{1}}, f_{\bar{2}}, f_1$ orthogonally, compare Fig. 10, left. Consequently, the four points $\iota (s_1)$, $\iota (s_{\bar{1}})$, $\iota (s_2)$, and $\iota (s_{\bar{2}})$ all lie in the subspace $\iota (s)^\perp \cap \iota (\epsilon )^\perp$. Its dimension is two, since $\iota (\epsilon )$ and $\iota (s)$ are linearly independent. Hence, $\iota (s)$ is a pseudo-principal checkerboard pattern in ${\mathbb {P}}{\mathbb {R}}^{4,1}$.

Now let $g$ be a pseudo-principal net in ${\mathbb {P}}{\mathbb {R}}^{4,1}$ and let *U* be the two-dimensional projective subspace that contains the four vertices $g_{1}$, $g_{\bar{1}}$, $g_2$, and $g_{\bar{2}}$. We denote by $U^\perp$ its orthogonal complement with respect to the Minkowski inner product $\langle \!\langle \,{\cdot },\,{\cdot }\,\rangle \!\rangle$. The space of all points in ${\mathbb {P}}{\mathbb {R}}^{4,1}$ that represent a plane in ${\mathbb {R}}^3$ is given by $\{\textbf{e}_{\infty }\}^\perp$. Referring to the projective space ${\mathbb {P}\mathbb {R}^{4,1}}$ we have $\dim U^\perp = 1$ and $\dim {\{\textbf{e}_{\infty }\}^\perp }=3$. It follows that $\dim {( U^\perp \!\cap \{\textbf{e}_{\infty }\}^\perp )} \geqslant 0$ and thus contains at least one point $\epsilon$. Since $\epsilon$ is a plane that intersects all points in *U* orthogonally, we conclude that all centers of $g_{1}$, $g_{\bar{1}}$, $g_2$, and $g_{\bar{2}}$ lie in $\epsilon$ and thus $\iota ^{-1}(g)$ is a principal sphere congruence. $\square$

Now Theorem 4.4 easily follows from Theorem 4.7.

#### Proof of Theorem 4.4

**Fig. 11:**
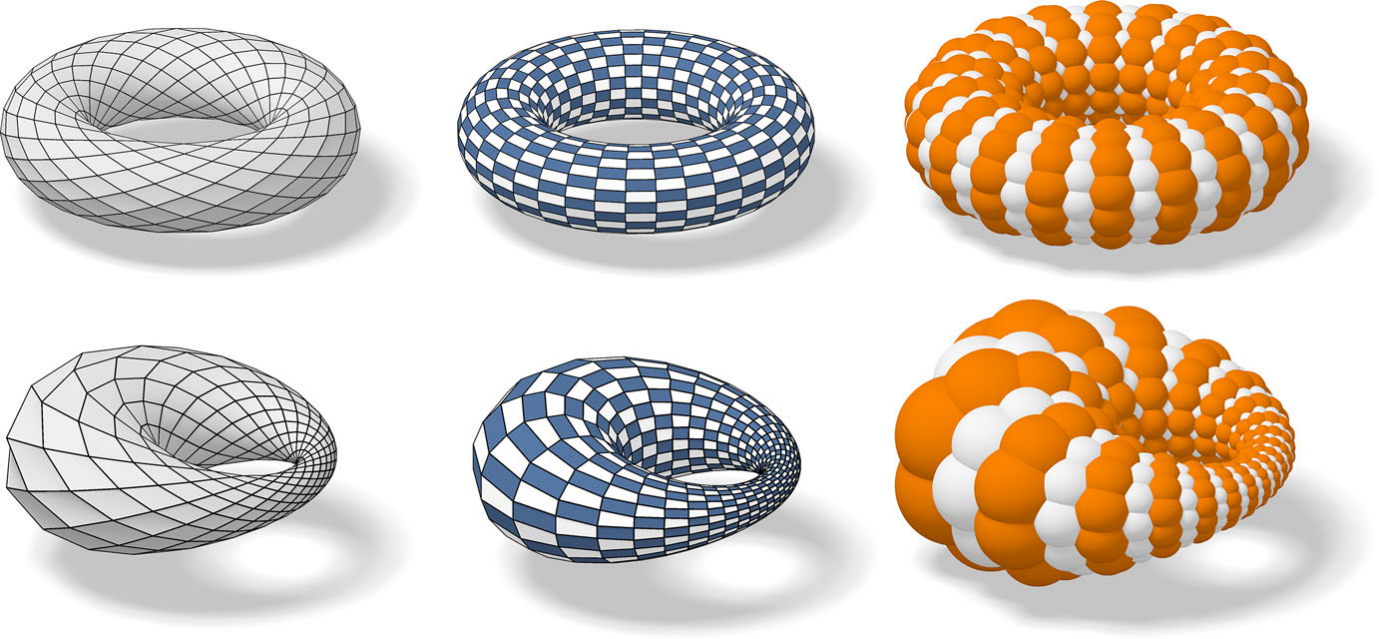
In the first row we see, from left to right, the control net, the checkerboard pattern and a Möbius representation of a principal net on the torus. The second row shows the image of the first row after a Möbius transformation is applied to the Möbius representation

As Möbius transformations in ${\mathbb {P}\mathbb {R}^{4,1}}$ are given by orthogonal transformations of ${\mathbb {R}}^{4,1}$, they preserve both orthogonality and *k*-dimensional subspaces. Thus pseudo-principal nets are mapped to pseudo-principal nets in ${\mathbb {P}\mathbb {R}^{4,1}}$ and by Theorem 4.7 this translates to principal nets in ${\mathbb {R}}^3$ as well. The application of Theorem 4.4 is demonstrated in Fig. 11. $\square$

#### Remark 4.8

In [19] an *n*-dimensional generalization of orthogonal checkerboard patterns is discussed. The three-dimensional case would be a pair of nets $D_1,D_2:{\mathbb {Z}}^3\rightarrow {\mathbb {R}}^n$, where an edge of $D_1$ is orthogonal to all edges of the corresponding face of $D_2$ and vice versa. Consequently, every face is planar which makes every two-dimensional cut of the pair $D_1,D_2$ a pair of diagonal nets of a principal checkerboard pattern in our sense. Thus, the preservation of planarity is an obvious consequence of the preservation of orthogonality from a higher-dimensional point of view.

#### Remark 4.9

In classical differential geometry, a principal net *f* can be characterized by the fact that its lift to the light cone $\hat{f}=f+\textbf{e}_0+|f|^2\textbf{e}_{\infty }$ is a conjugate net. The mapping $\iota (\,{\cdot }\,)$ is a natural discretization of $f\mapsto \hat{f}$ as $\iota (s)$ converges to $\hat{f}$ if the radius of the sphere *s* with center *f* converges to zero. Like in the classical theory $\iota (s)$ is a conjugate net. However, $\iota (s)$ reveals even more structure, namely the orthogonality of spheres, that cannot be observed in the limit anymore.

### A Projective Point of View

It is enlightening to also study the embedding of the sphere congruence to ${\mathbb {P}\mathbb {R}^{4,1}}$ from a more geometric perspective.

**Fig. 12:**
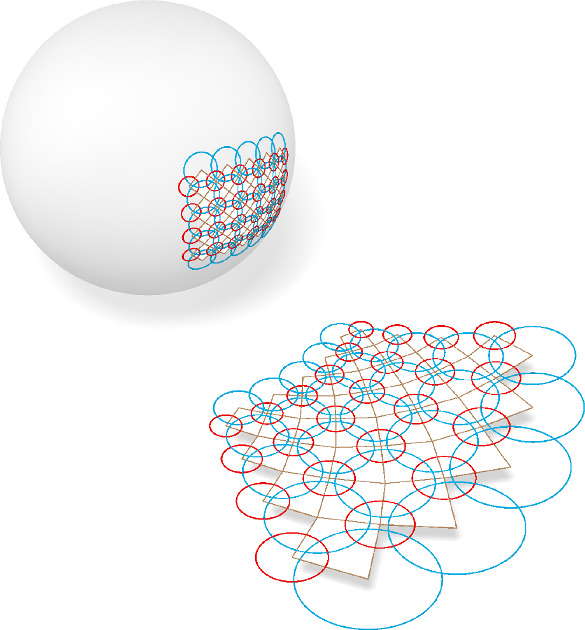
The geometric description of the mapping $\iota$ in ${\mathbb {R}}^2$. A planar orthogonal circle pattern is stereographically projected onto the unit sphere. A new orthogonal net in space is obtained by the polar points of the circles on the sphere

The mapping $\iota$ can be seen as stereographically projecting a sphere *s* to the unit sphere ${\mathbb {S}}^3$ and further mapping the image $s' \subseteq {\mathbb {S}}^3$ to its polar point $p=\iota (s)$ with respect to ${\mathbb {S}}^3$, compare Fig. 12. The polar point *p* is the apex point of the cone that touches ${\mathbb {S}}^3$ along $s'$. The polar point of any sphere $s_1'\subseteq {\mathbb {S}}^3$ that intersects $s'$ orthogonally lies in the polar hyperplane of *p* and is thus conjugate to *p*. Hence, the diagonals of the quadrilateral $(\iota (s), \iota (s_1), \iota (s_{12}), \iota (s_2))$ are not only orthogonal but conjugate with respect to ${\mathbb {S}}^3$. The projective approach also gives meaning to the vertices of $f$ in the projective model. They are the images of $\iota (s)$ under the central projection ${\mathbb {R}}^4 \rightarrow {\mathbb {R}}^3$ through the north pole of ${\mathbb {S}}^3$.

#### Remark 4.10

The unique sphere with center $\iota (s)$ that intersects ${\mathbb {S}}^3$ orthogonally, intersects ${\mathbb {S}}^3$ along $s'$. Hence, the vertices $\iota (s_{f})$ define a unique sphere congruence $\mathcal {S}$ of three-dimensional spheres, where every sphere intersects its neighbors and also ${\mathbb {S}}^3$ orthogonally. The stereographic projection ${\mathbb {S}}^3 \rightarrow {\mathbb {R}}^3$ can be extended to a Möbius transformation $\zeta :{\mathbb {P}\mathbb {R}^{4,1}}\rightarrow {\mathbb {P}\mathbb {R}^{4,1}}$. The spheres of $s_{f}$ can be directly obtained from the spheres of $\mathcal {S}$ by applying $\zeta$ and then intersecting the image with ${\mathbb {R}}^3$.

#### Remark 4.11

**Fig. 13:**
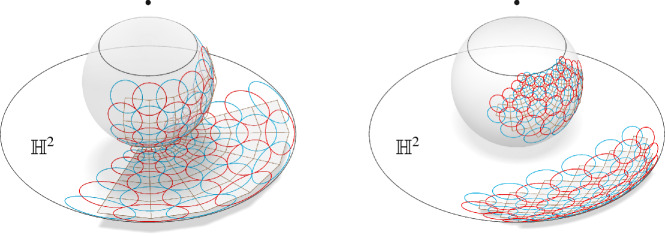
Both images show an orthogonal net of circles on ${\mathbb {S}}^2$ which by a central projection is mapped to a net of conics. The net of conics is an h-orthogonal net of h-circles in the Cayley–Klein model of hyperbolic geometry

This geometric approach further allows us to generalize orthogonal sphere congruences to non-Euclidean geometry by replacing the stereographic projection from ${\mathbb {S}}^3$ to ${\mathbb {R}}^3$ by a central projection $\psi :{\mathbb {S}}^3 \rightarrow {\mathbb {R}}^3$. A sphere congruence on ${\mathbb {S}}^3$ conjugate with respect to ${\mathbb {S}}^3$ gets mapped to a congruence of non-Euclidean spheres. These non-Euclidean spheres intersect in directions conjugate with respect to $\psi ({\mathbb {S}}^3)^*$ the contour quadric of $\psi ({\mathbb {S}}^3)$, compare Fig. 13 and Lemma B.1 in Appendix B.

### A Gauß Image for Principal Nets

As mentioned in Remark 3.11, we can find an alternative definition of a Gauß image making use of the polarity properties of principal nets. This alternative is particularly interesting in connection with the minimal surfaces described in Sect. 6.1.

#### Definition 4.12

If $f$ is the control net of a principal checkerboard pattern $c_{f}$, then *n* is a *principal Gauß image* of $f$ and $c_{f}$, if the edges of $c_{f}$ are parallel to the edges of $c_{n}$ and every sphere of $s_{n}$ intersects the unit sphere orthogonally.

The principal Gauß image *n* of $f$ from Definition 4.12 can be seen as a parallel net of $f$ on the unit sphere. The parallelism can be observed in the corresponding checkerboard patterns $c_{f}$ and $c_{n}$, while the connection to the unit sphere can be observed in the Möbius representation $s_{n}$. Instead of requiring vertices to lie exactly on the unit sphere, we require their corresponding spheres to intersect the unit sphere orthogonally. In the limit of spheres with radius zero the vertices lie exactly on the unit sphere. The principal Gauß image of a principal net $f$ is determined up to the choice of one initial vertex along a prescribed line.

#### Lemma 4.13

Let $f$ be a net with principal checkerboard pattern. Then there exists a one parameter family of principal Gauß images *n* of $f$ in the sense of Definition 4.12. Diagonals of faces of *n* are polar to one another with respect to the unit sphere.

#### Proof

To show the polarity of diagonals we consider a quadrilateral of four spheres $(s,s_1,s_{12}, s_2)$ with centers $(n,n_1,n_{12},n_2)$ that intersect ${\mathbb {S}}^2$ orthogonally. Additionally every sphere intersects its neighbors orthogonally. The centers of all spheres that intersect both ${\mathbb {S}}^2$ and *s* orthogonally lie on a plane that contains the circle ${\mathbb {S}}^2 \cap s$. This plane is exactly the polar plane of *n*. The same argument goes for $n_{12}$ and thus the diagonals $(n_1,n_2)$ and $(n,n_{12})$ lie on conjugate lines.

The uniqueness follows immediately from the polarity. Let us fix one vertex *n*(*k*, *l*) of *n*. Due to the parallelism of checkerboard patterns, we know the directions of diagonals emanating from *n*(*k*, *l*). The four corresponding polar lines all lie in the polar plane of *n*(*k*, *l*) and their intersection points determine the neighbors of *n*(*k*, *l*). Thus, the initial vertex *n*(*k*, *l*) corresponding to *f*(*k*, *l*) needs to be chosen on a line orthogonal to $\mathcal {W}_{f}(k,l)$. Note that polar lines are orthogonal and thus the parallelism is preserved. As polarity is a symmetric relation this process can be extended over the entire net.

Now we can choose the initial radius of the sphere *s*(*k*, *l*) at vertex *n*(*k*, *l*) such that it intersects ${\mathbb {S}}^2$ orthogonally. The neighboring spheres of *s*(*k*, *l*) have their centers in the plane of all centers of spheres that intersect *s*(*k*, *l*) and ${\mathbb {S}}^2$ orthogonally. Hence, all radii can be chosen such that the orthogonal intersection with both, all neighbors and the unit sphere is met. Hence the so constructed net *n* is indeed the principal Gauß image of $f$. $\square$

#### Remark 4.14

We could also use the principal Gauß image in Definition 3.3 of the shape operator. This only works for principal nets but it allows us to drop the orthogonal projection $P_N$. Moreover, this approach fits the theory of minimal surfaces very well, as we will discuss in Sect. 6.1.

## Koenigs Nets

In [7] Adam Doliwa defined discrete Koenigs nets as those conjugate nets where for every quadrilateral the six focal points lie on a common conic section, the so called conic of Koenigs. We apply the same definition to the checkerboard pattern $c_{f}$ instead of the control net $f$ obtaining thus a subclass of the nets defined in [7]. This adaptation proves to be very useful as we can naturally dualize checkerboard patterns. Analogous to the smooth theory such a dual checkerboard pattern exists if and only if $c_{f}$ is a Koenigs net. Even though the definition of Koenigs nets is based on checkerboard patterns we find that the class of Koenigs nets is invariant under projective transformations applied to the vertices of the corresponding control nets. Again in [7], Doliwa defined discrete analogs of the so called Laplace invariants of a conjugate net. These projective invariants appear, in a slightly adapted way, in the checkerboard approach as well. However, it is only in this setting that Koenigs nets can be characterized as exactly those nets that have equal Laplace invariants analogously to the smooth theory.

### Characterization of Koenigs Nets

The discretization in both this paper and in [7] is based on the smooth characterization of Koenigs nets that can be found in [13].

#### Definition 5.1

**Fig. 14:**
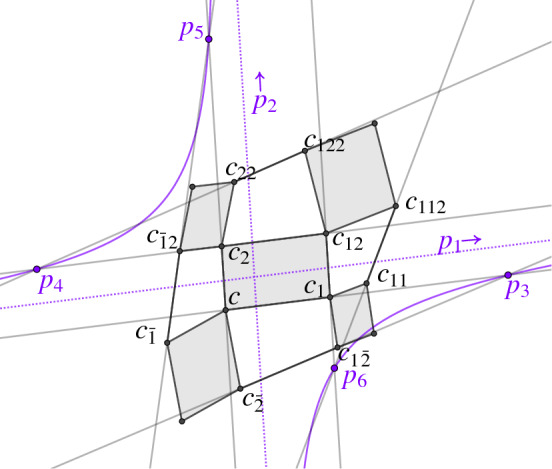
Definition of a Koenigs net. The supporting lines of neighboring edges in the checkerboard pattern intersect in the six points $p_1,\dots ,p_6$. If all of them lie on a common conic section the checkerboard pattern is Koenigs. The points $p_1$ and $p_2$ are always at infinity, here indicated by the dotted line, so the conic section is a hyperbola

Let *c* be a conjugate checkerboard pattern. For the edge $(c,c_i)$ we denote the supporting line by $\ell (c,c_i)$. We call the checkerboard pattern *c* a *Koenigs checkerboard pattern* if for every first order face $(c, c_1, c_{12}, c_2)$ the six points

$$\begin{aligned}&p_1 = \ell (c,c_1) \cap \ell (c_2,c_{12}),&p_2 = \ell (c,c_2) \cap \ell (c_1,c_{12}), \\&\quad \,\, p_3= \ell (c,c_1) \cap \ell (c_{-2},c_{1-2}),&p_4 = \ell (c_2,c_{12}) \cap \ell (c_{22},c_{122}), \\&p_5 =\ell (c,c_2) \cap \ell (c_{\bar{1}},c_{\bar{1}2}),&p_6 =\ell (c_1,c_{12}) \cap \ell (c_{11},c_{112}), \end{aligned}$$

are all different and lie on a common conic section, see Fig. 14.

#### Remark 5.2

Since in Definition 5.1 the points $p_1$ and $p_2$ are always at infinity, we know that the conic of Koenigs is always a hyperbola. Around every second order face the existence of a conic is always met, as the four points $p_3,\dots ,p_6$ lie on the line at infinity. Thus every Koenigs checkerboard pattern is also a Koenigs net in the sense of [7].

Analogously to [5], the Koenigs property is equivalent to the closeness of a multiplicative one-form defined on the edges of the checkerboard pattern.

#### Definition 5.3

**Fig. 15:**
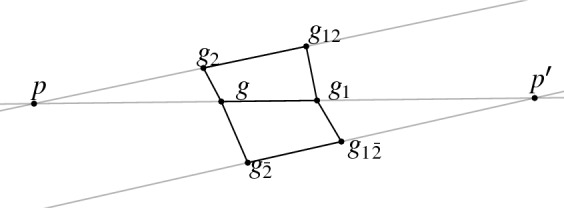
The multiplicative one-form *q* is defined on the edge $(g,g_1)$ as

(*multiplicative one-form*)  Let *g* be a net with planar quadrilaterals. Let further $p = \ell (g,g_1) \cap \ell (g_2,g_{12})$ and $p' = \ell (g,g_1) \cap \ell (g_{\bar{2}},g_{1\bar{2}})$, see Fig. 15. We define a multiplicative one-form *q* along the edge $(g,g_1)$ as the cross-ratio of the four points *g*, $g_1$, *p*, and $p'$,and along the edge $(g_1,g)$ asAnalogously we define $q(g,g_2)$ and $q(g_2,g)$ aswhere $r=\ell (g,g_2) \cap \ell (g_{\bar{1}},g_{\bar{1}2})$ and $r'= \ell (g,g_2) \cap \ell (g_{1},g_2)$.

#### Remark 5.4

The multiplicative one-form from Definition 5.3 is known in the literature by the name Laplace-Invariant, see for example [4, p. 77, Exer. 2.15]. However, in this paper the name Laplace invariant is reserved for Definition 5.7, where we basically apply the same definition to the edges of the control net.

The next theorem is contained in [4] as Exercise 2.23. It could also be formulated for general quadrilateral nets with planar faces.

#### Theorem 5.5

Let *c* be a conjugate checkerboard pattern of disc-topology such that around every first order face the six points $p_1,\dots ,p_6$ from Definition 5.1 are all distinct. Let further *q* be the multiplicative one-form from Definition 5.3 defined on the edges of $c$. Then *q* is closed if and only if *c* is Koenigs.

#### Proof

This theorem can be proven by introducing a projective coordinate system followed by lengthy computations that can be found in detail in the appendix or in [4, p. 374]. $\square$

The multiplicative one-form can also be formulated via the vertices of the control net as the following lemma shows.

#### Lemma 5.6

**Fig. 16:**
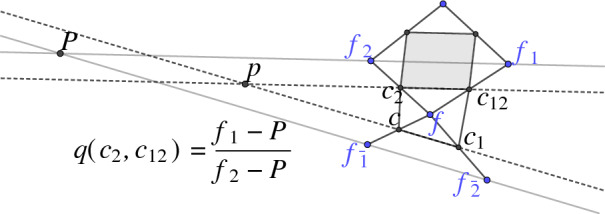
The one-form *q* defined on the edges of the checkerboard pattern can be expressed by the vertices of the control net

Let $\mathcal {W}_{f} = (c,c_1,c_{12},c_2)$ be a second order face of a conjugate checkerboard pattern *c* with control net *f*. We choose the notation such that $c = (f_{\bar{1}} + f)/2$, $c_1 = (f + f_{\bar{2}})/2$, $c_{12} = (f + f_1)/2$ and, $c_2 = (f + f_2)/2$, see Fig. 16. Let further $p = \ell (c_2,c_{12}) \cap \ell (c,c_1)$ and $P = \ell (f_{\bar{1}},f_{\bar{2}}) \cap \ell (f_1,f_2)$. Then the multiplicative one-form *q* is computed on the edge $(c_2, c_{12})$ as

#### Proof

First note that the point $p' = \ell (c_2,c_{12}) \cap \ell (c_{22},c_{122})$ lies at infinity. Thus the fraction $({c_2 - p'})/({c_{12} - p'})$ in the definition of $q(c_2,c_{12})$ equals 1. Since the quadrilateral $(f_{\bar{1}}, f_{\bar{2}}, f_{1}, f_{2})$ is the image of the quadrilateral $(c,c_1,c_{12},c_2)$ under the affine mapping $\alpha (x) = 2x - f$ the second equality holds. $\square$

This gives rise to the following lemma and definition.

**Fig. 17:**
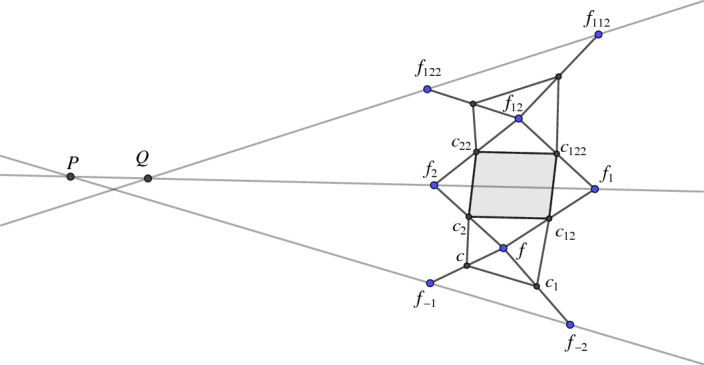
The product  equals the cross-ratio  and is thus invariant under projective transformations applied to the control net

#### Lemma and Definition 5.7

Consider the setting of Fig. 17 with the first order face $(c_2, c_{12}, c_{122}, c_{22})$. The product  is a projective invariant of the control net. It is called *Laplace invariant* and can be expressed via the control net by6For every face of the control net we have two Laplace invariants associated with the corresponding diagonals.

#### Theorem 5.8

Let $c_{f}$ be a conjugate checkerboard pattern with control net $f$ such that for every face the six points $p_1,\dots ,p_6$ from Definition 5.1 are all distinct. Then $c_{f}$ is Koenigs if and only if for every face of *f* the two Laplace invariants associated to its diagonals are equal.

#### Proof

The two Laplace invariants of a face of the control net are equal if and only if the multiplicative one-form defined on the edges of the inscribed first order face is closed. Hence the statement follows from Theorem 5.5. $\square$

#### Remark 5.9

There are special cases where not all points $p_1,\dots ,p_6$ are distinct, but the Laplace invariants are still equal. Those cases will turn out to be dualizable as well, so it makes sense to consider these nets to be Koenigs nets as well.

#### Remark 5.10

It is worth noticing that Theorem 5.8 is independent of the choice of the control net. So if a checkerboard pattern is Koenigs every associated control net has equal Laplace invariants.

#### Corollary 5.11

Koenigs checkerboard patterns are mapped to Koenigs checkerboard patterns under projective transformations applied to the vertices of the control nets.

#### Proof

The Laplace invariants are defined as cross-ratios of vertices and intersection points of lines of the control net. Hence it is invariant under projective transformations and so the property of equal invariants is preserved as well. $\square$

#### Remark 5.12

Discrete Laplace invariants are defined for Koenigs nets in [7, p. 5] in a similar fashion. The benefit of the checkerboard pattern approach is that now the Koenigs nets can be characterized as “nets with equal invariants”, compare Theorem 5.8, like one would expect coming from the smooth theory.

### Dualization

#### Definition 5.13

Let $c$ be a checkerboard pattern. We call $c'$ a *dual checkerboard pattern* of $c$, if it is edgewise parallel and corresponding first order faces are similar but have reversed orientation. If such a dual checkerboard pattern $c'$ exists, we call $c$ *dualizable*.

In analogy to the smooth case we find that the dualizable checkerboard patterns are precisely the Koenigs checkerboard patterns. The following theorem holds.

#### Theorem 5.14

**Fig. 18:**
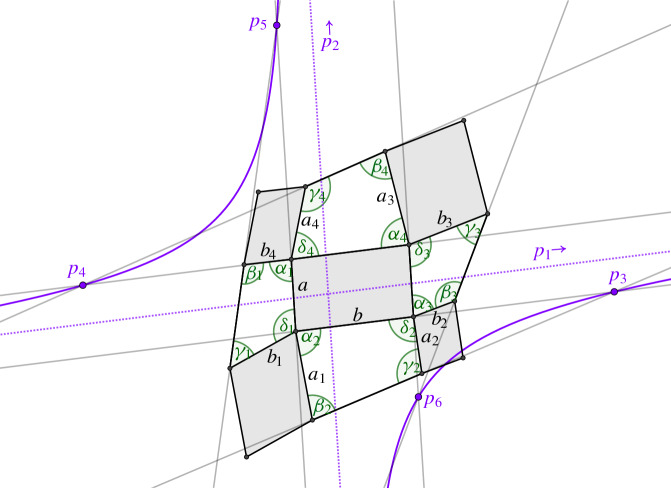
The configuration of Theorem 5.14

Let $c$ be a conjugate checkerboard pattern. We introduce the following local notation in the face patch of a given first order face, see Fig. 18:

- Let $a = \Vert \delta _v f\Vert$ and $b = \Vert \delta _u f\Vert$ be the edge lengths of the central first order face.
- We enumerate the surrounding first order faces counterclockwise and denote their edge lengths with $a_i$ and $b_i$ accordingly.
- For every second order face in the patch we denote its interior angles by $\alpha _i$, $\beta _i$, $\gamma _i$, and $\delta _i$ in counterclockwise order.

Let $r_i = {a_i}/{b_i}$ be the ratio of edge lengths for each first order face. If no two of the six points $p_1,\dots , p_6$ from Definition 5.1 are equal, the following conditions are equivalent:

(i).
(ii) *There exists a non-trivial conformal Combescure transformation of* $c$. *This means that a checkerboard pattern with parallel edges exists where corresponding first order faces differ only by a similarity transformation. If there is one such transformation, then there exists an entire two-parameter family of such transformations.*
(iii) $c$ *is dualizable.*
(iv) $c$ *is a Koenigs checkerboard pattern.*

#### Proof

First we show (i) $\Leftrightarrow$ (ii). If *j* is even, the edge lengths $a_{j-1}$ and $a_{j}$ are related by the formula

$$\begin{aligned}a_{j-1} = k_j a_j + c_j b,\end{aligned}$$

whereAnalogously we find $b_2 = k_3 b_3 + c_3 a$ and $b_4 = k_1 b_1 + c_1 a$. Using $a_i = b_i r_i$ we find the closing conditionHence we can compute $a_1$ only from the given angles and ratios if and only if7Consequently a non-trivial parallel net with the same ratios *r* and $r_i$ exists if and only if (i) holds.

Next we show that (i) $\Leftrightarrow$ (iii) holds. When we dualize the net $f$ all angles are replaced by their respective complementary angles, i.e., $\alpha _i^* = \pi - \alpha _i$, $\beta _i^* = \pi - \beta _i$, $\gamma _i^* = \pi - \gamma _i$, and $\delta _i^* = \pi - \delta _i$. Hence the coefficients $k_i$ are invariant under dualization while the coefficients $c_i$ change sign. If we denote the transformed edge lengths by $a_i^*, a^*$ and $b_i^*, b^*$ respectively, then the transformed relations read

$$\begin{aligned}a_{j-1}^* = k_j a_j^* - c_jb^* \ \ \quad \text {and}\ \ \quad b_{j-1}^*= k_j b_j^* - c_j a^*.\end{aligned}$$

So the closing condition becomesAgain we find that $a_1^*$ can be determined from this equation if (7) holds. However, comparing the potential formulas for $a_1$ and $a_1^*$ we find that $a_1^* = - a_1$. As no negative edge lengths can exist we conclude that $a_1^*$ exists only if (i) holds. On the other hand if (i) holds, we can construct a dual net for any value $a_1^*$ implying (iii).

Next we show (i) $\Leftrightarrow$ (iv). To do so we use the inscribed angle theorem for hyperbolas (see Theorem B.2 in the appendix). Let $k(\ell (p_i,p_j))$ denote the slope of the line $\ell (p_i,p_j)$ with respect to a coordinate system aligned with the asymptotes of the hyperbola, compare Theorem B.2. We find 
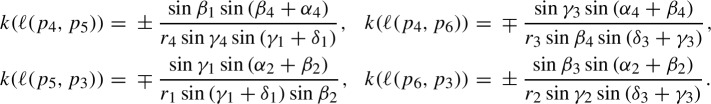


Note that  is equivalent to $\ell (c_{\bar{1}},c_{\bar{1}2}) \,{\parallel }\, \ell (c,c_2)$ which is further equivalent to $p_5 = p_2$. So if the points $p_1,\dots ,p_6$ are all distinct, the denominators in the above equations are all nonzero. Computing the quotients yieldsBy Theorem B.2 the points $p_1,\dots ,p_6$ lie on a common hyperbola if and only if

$$\begin{aligned}\frac{r_2}{r_1}\cdot \frac{\sin \gamma _1\sin \gamma _2}{\sin \beta _2\sin \beta _3}=\frac{r_3}{r_4}\cdot \frac{\sin \beta _1\sin \beta _4}{\sin \gamma _3\sin \gamma _4}\end{aligned}$$

which is equivalent to (i). This concludes the proof. $\square$

#### Remark 5.15

If we find $p_i = p_j$ for some $i \ne j$ everything in the proof of Theorem 5.14 still holds except for the application of Theorem B.2. So in such a case we still find that (i) $\Leftrightarrow$ (ii) $\Leftrightarrow$ (iii).

#### Corollary 5.16

Let $c_{f}$ be a conjugate checkerboard pattern with control net $f$. Then $c_{f}$ is dualizable if and only if each two Laplace invariants defined in the faces of $f$ are equal.

#### Proof

If the six points $p_1,\dots ,p_6$ from Definition 5.1 are distinct, the statement follows from Theorem 5.14. Hence condition (i) in Theorem 5.14 is equivalent to the multiplicative one-form *q* being closed if $p_1,\dots ,p_6$ are all distinct. However these terms depend continuously on the vertices of the checkerboard pattern. Hence, any face patch, on which *q* is closed, can be approximated with a sequence of dualizable face patches where $p_1,\dots ,p_6$ are distinct. Since condition (i) is preserved in the limit, so is the existence of a dual. $\square$

#### Remark 5.17

**Fig. 19:**
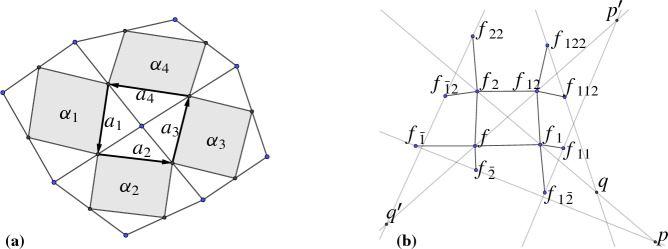
Left: The idea behind an efficient implementation of a dualization algorithm. The edges $a_i$ have to close in the initial net as well as in the dualized net. From this condition the scaling factors that guide the dualization can be computed. Right: The setting of Lemma 5.18

For a given Koenigs checkerboard pattern, there is a two-parameter family of dual checkerboard patterns that differ in the scaling of corresponding first order faces. By choosing the initial scaling factors of two adjacent first order faces $\alpha _1$ and $\alpha _2$, all other scaling factors can be computed recursively by the formulaswhere $a_i$ are the oriented edges of the corresponding first order faces, see Fig. 19, left. This permits a stable dualization algorithm.

The following lemma provides an easy way to generate a specific family of Koenigs nets in ${\mathbb {P}}{\mathbb {R}}^2$.

#### Lemma 5.18

Let *M* and *N* be two commuting projective transformations ${\mathbb {P}}{\mathbb {R}}^2 \rightarrow {\mathbb {P}}{\mathbb {R}}^2$ and let $P\in {\mathbb {P}}{\mathbb {R}}^2$. Then the net $f$ defined by $f(k,l) = M^k N^l P$ is the control net of a Koenigs checkerboard pattern in ${\mathbb {P}}{\mathbb {R}}^2$.

#### Proof

We show that the condition of Theorem 5.8 is met in the quadrilateral $(f, f_{1}, f_{12}, f_{2})$, compare Fig. 19, right. Let 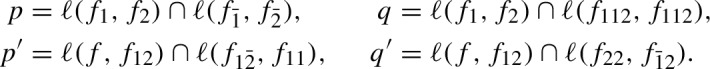


Let $F:{\mathbb {Z}}^2 \rightarrow {\mathbb {R}}^3$ be the net of homogeneous coordinates of the vertices of $f$. We findFrom this we can formulate the cross-ratios asNow let $\textbf{M}$ and $\textbf{N}$ be the matrix representations of *M* and *N* in homogeneous coordinates. Then we can express the cross-ratios asSo we see that the two Laplace invariants  and  are equal. $\square$

### Connection to the Existing Theory

Besides the definition of Koenig nets given in [7] an elegant and popular discretization of Koenigs nets can be found in [5]. There Koenigs nets are defined as nets with planar quadrilaterals that admit a dual net. Two quadrilaterals are considered dual if they have parallel edges and non-corresponding diagonals are parallel. Consequently two quadrilateral nets are dual if all corresponding quadrilaterals are dual. These Koenigs nets have several interesting properties summarized in [4, Chapter 2.3] and like in this paper they can be used to characterize discrete isothermic nets which results in the class of isothermic nets defined in [1].

**Fig. 20:**
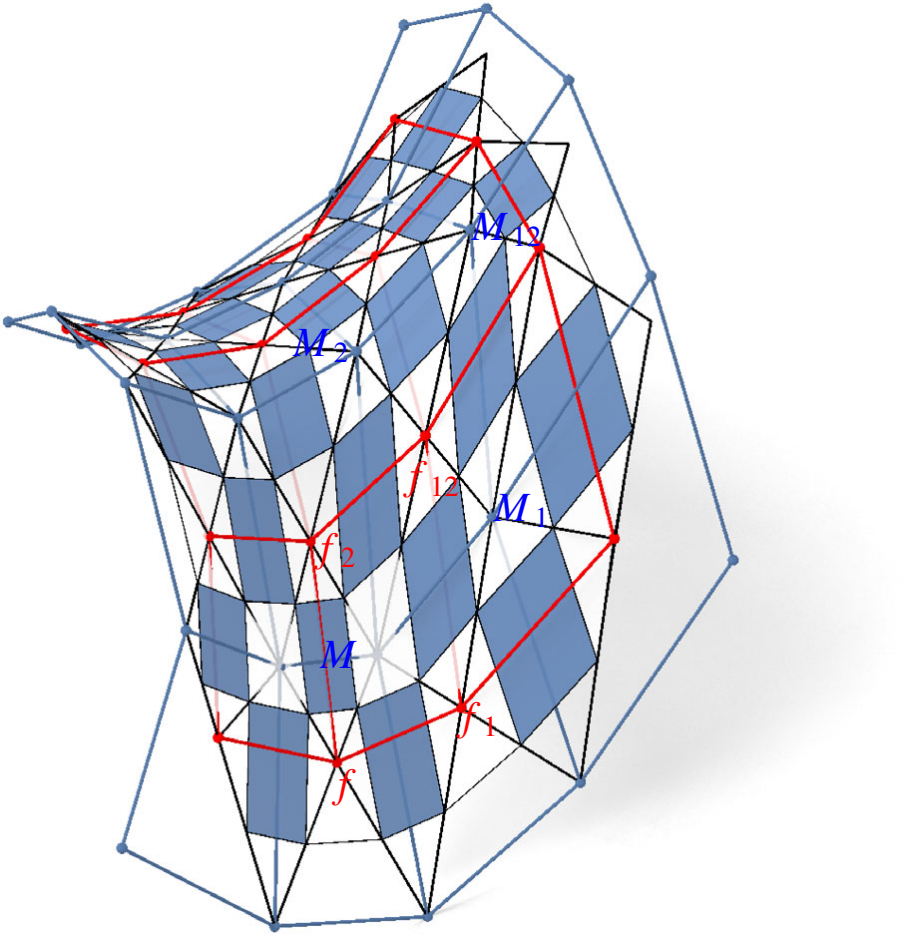
The intersection points of diagonals of a Koenigs net in the sense of [5] (blue) form a Koenigs net in the sense of [7] (red). The Laplace invariant of the edge $(f,f_1)$ equals the Laplace invariant of the edge $(M_{\bar{2}},M)$. Consequently the control net with the faces $(f,M_{\bar{2}},f_1,M)$ and $(f_1,M_1,f_{12},M)$ is the control net of a Koenigs checkerboard pattern in the sense of Definition 5.1

**Fig. 21:**
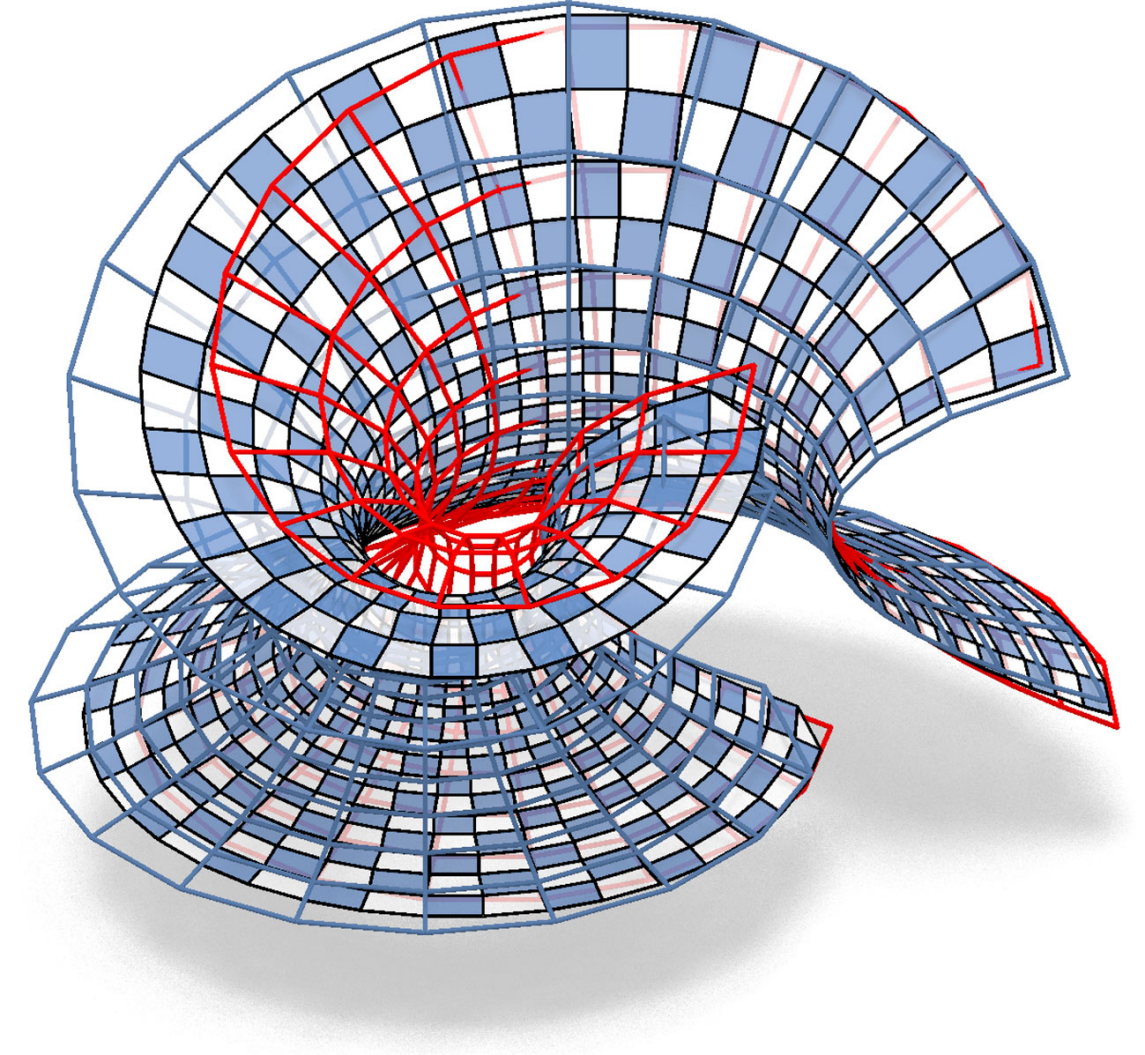
A Koenigs checkerboard pattern and the corresponding diagonal nets. It is constructed by applying a Möbius transformation to an isothermic net in the plane followed by dualization, compare Sect. 6.1. Neither of the diagonal nets is Koenigs in the sense of [5] or [7]

The author is grateful to Jan Techter for the following observation. The Koenigs nets defined in this paper are in some sense a generalization of the Koenigs nets defined in [5]. As a Remark in [5, p. 15] says, the intersection points *M* of diagonals of the faces of a [5]-Koenigs net form a Koenigs net in the sense of [7], compare Fig. 20. A proof for this remark can be found in [4, pp. 373–374]. Moreover this proof reveals that the Laplace invariant of the edge $(f,f_1)$ is equal to the Laplace invariant of the edge $(M_{-2},M)$. Hence the two Koenigs nets together form a control net of a Koenigs checkerboard net, where the faces are given by $(f,M_{-2},f_1,M)$ and $(f_1,M_1,f_{12},M)$. This means that every Koenigs net in the sense of [5] can be extended to a Koenigs checkerboard net according to Definition 5.1. The converse is not true as a numerical analysis of the Koenigs checkerboard pattern in Fig. 21 shows.

#### Remark 5.19

The connection to Koenigs nets in the sense of [5] offers a way to construct non-planar Koenigs nets. Another way to generate examples of such Koenigs nets will be presented in the next section through Theorem 6.3 and Corollary 6.2.

## Isothermic Nets

Discrete isothermic nets can now be defined as principal nets that are also Koenigs nets, similarly to [2, 3, 5]. Analogous to the smooth case or to other discrete approaches [5] we find that the class of discrete isothermic nets is invariant under dualizations and Möbius transformations. This permits a construction of discrete minimal surfaces and their Goursat transformations as will be described later on.

### Definition 6.1

We call a checkerboard pattern $c$ *isothermic*, if it is both principal and Koenigs.

**Fig. 22:**
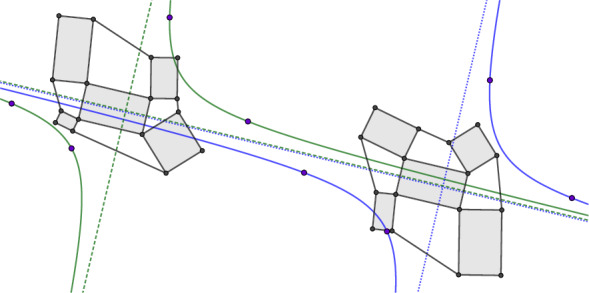
An isothermic checkerboard pattern and its dual with the corresponding conics of Koenigs. The points on the hyperbolas are the points of intersecting supporting lines of neighboring edges

As orthogonal first order faces are mapped to orthogonal faces under dualization, the next corollary follows immediately from Theorem 5.14. See Fig. 22 for an illustration.

### Corollary 6.2

Isothermic checkerboard patterns are dualizable. Their dual is again an isothermic checkerboard pattern.

### Theorem 6.3

**Fig. 23:**
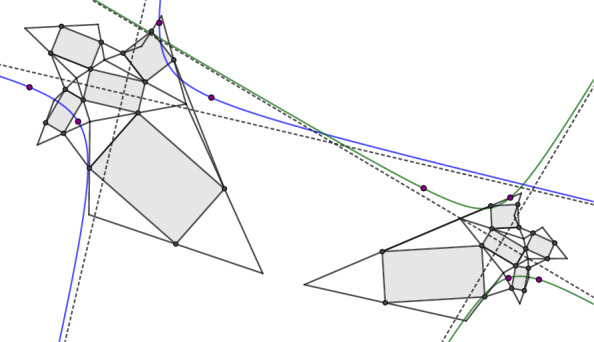
An isothermic checkerboard pattern and its Möbius transform together with the corresponding conics of Koenigs. The figure features non-convex quads as the examples were constructed in such a way that the points of intersecting lines are all close to the checkerboard pattern

(Möbius invariance)   Isothermic checkerboard patterns are mapped to isothermic checkerboard patterns under a discrete Möbius transformation, see Fig. 23.

The proof is a direct consequence of Lemma 6.5 and is thus postponed for now. In order to prove Theorem 6.3, we study isothermic nets again in the space ${\mathbb {P}\mathbb {R}^{4,1}}$ under the embedding $\iota$. We have already defined pseudo-principal nets in ${\mathbb {P}\mathbb {R}^{4,1}}$ and can now extend them to pseudo-isothermic nets.

### Definition 6.4

We call a net $g$ in ${\mathbb {P}\mathbb {R}^{4,1}}$ pseudo-isothermic if it is pseudo-principal and the two Laplace invariants for each face are equal.

It turns out that the lift $\iota (f)$ of an isothermic net $f$ in ${\mathbb {R}}^3$ is a pseudo-isothermic net in ${\mathbb {P}\mathbb {R}^{4,1}}$ as the following lemma shows.

### Lemma 6.5

Let $f$ be the control net of an isothermic checkerboard pattern and let $\iota (f)$ be its lift to ${\mathbb {P}\mathbb {R}^{4,1}}$. The Laplace invariants of corresponding faces of $f$ and $\iota (f)$ are equal.

### Proof

**Fig. 24:**
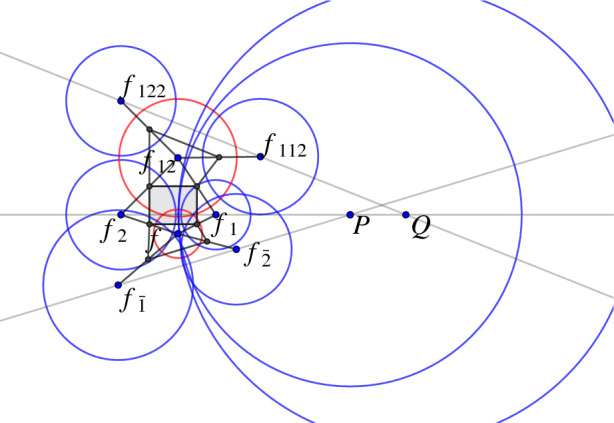
The idea behind the proof of Lemma 6.5: Not only do the lines $\ell (f_1,f_2)$ and $\ell (f_{\bar{1}},f_{\bar{2}})$ intersect in *P*, but also the corresponding pencils of spheres intersect in a sphere with center at *P*. This means that there is a sphere with center at *P* that intersects both the sphere with center at *f* and the sphere with center at $f_{12}$ orthogonally

First note that $\iota (f)$ has a conjugate checkerboard pattern and thus the Laplace invariants are well defined. Hence not only the supporting lines $\ell (f_{\bar{1}},f_{\bar{2}})$ and $\ell (f_1,f_2)$ intersect, but also the corresponding pencils of spheres, compare Fig. 24. However, we know that the first three components under the lift $\iota$ are the same as the original centers of spheres and when we compute the cross-ratio of points lying on a line it is sufficient to use just one coordinate. So it follows that the Laplace invariants remain unchanged under $\iota$. $\square$

From Lemma 6.5 the proof of Theorem 6.3 follows immediately.

### Proof of Theorem 6.3

Every Möbius transformation can be seen as a projective transformation in ${\mathbb {P}\mathbb {R}^{4,1}}$ that preserves the inner product. Obviously these transformations preserve the cross-ratio and since $\iota$ also preserves the Laplace invariants we can conclude that not only conjugacy and orthogonality, but also the Koenigs property is preserved under Möbius transformations. $\square$

### Minimal Surfaces

Minimal surfaces can be constructed by dualizing an isothermic net on the unit sphere, since the theory of minimal surfaces tells us that for any minimal surface its dual and its Gauß image are equal. With the Möbius transformation and dualization at hand we can reproduce this construction in the discrete setting.

#### Definition 6.6

Let $f$ be the control net of an isothermic checkerboard pattern $c_{f}$. We call $c_{f}$ minimal if it has a dual checkerboard pattern $c'$ that is also the checkerboard pattern of a principal Gauß image of *f* in the sense of Definition 4.12.

#### Definition 6.7

Let $f$ and $\tilde{f}$ be control nets of minimal checkerboard patterns. They are related by a *Goursat transformation* if their principal Gauß images are related by a Möbius transformation.

#### Definition 6.8

We say that a checkerboard pattern $c_{f}$ is *on the unit sphere*, if there is a Möbius representation $s_{f}$ where every sphere intersects the unit sphere orthogonally.

#### Corollary 6.9

Let $c_{n}$ be an isothermic checkerboard pattern on the unit sphere. The dual checkerboard pattern $c_{n}'$ is a minimal checkerboard pattern and *n* is its principal Gauß image. If *n* is used to compute the discrete shape operator of $c_{n}'$, the mean curvature of $c_{n}'$ is zero.

#### Proof

The first statement follows directly from the definition of minimal checkerboard patterns. The principal curvature $\kappa _1$ and $\kappa _2$ are just the oriented scaling factors between edges of $c_{n}$ and $c_{n}'$. If the Gauß image is the dual net at the same time the relation $\kappa _1=- \kappa _2$ holds. $\square$

**Fig. 25:**
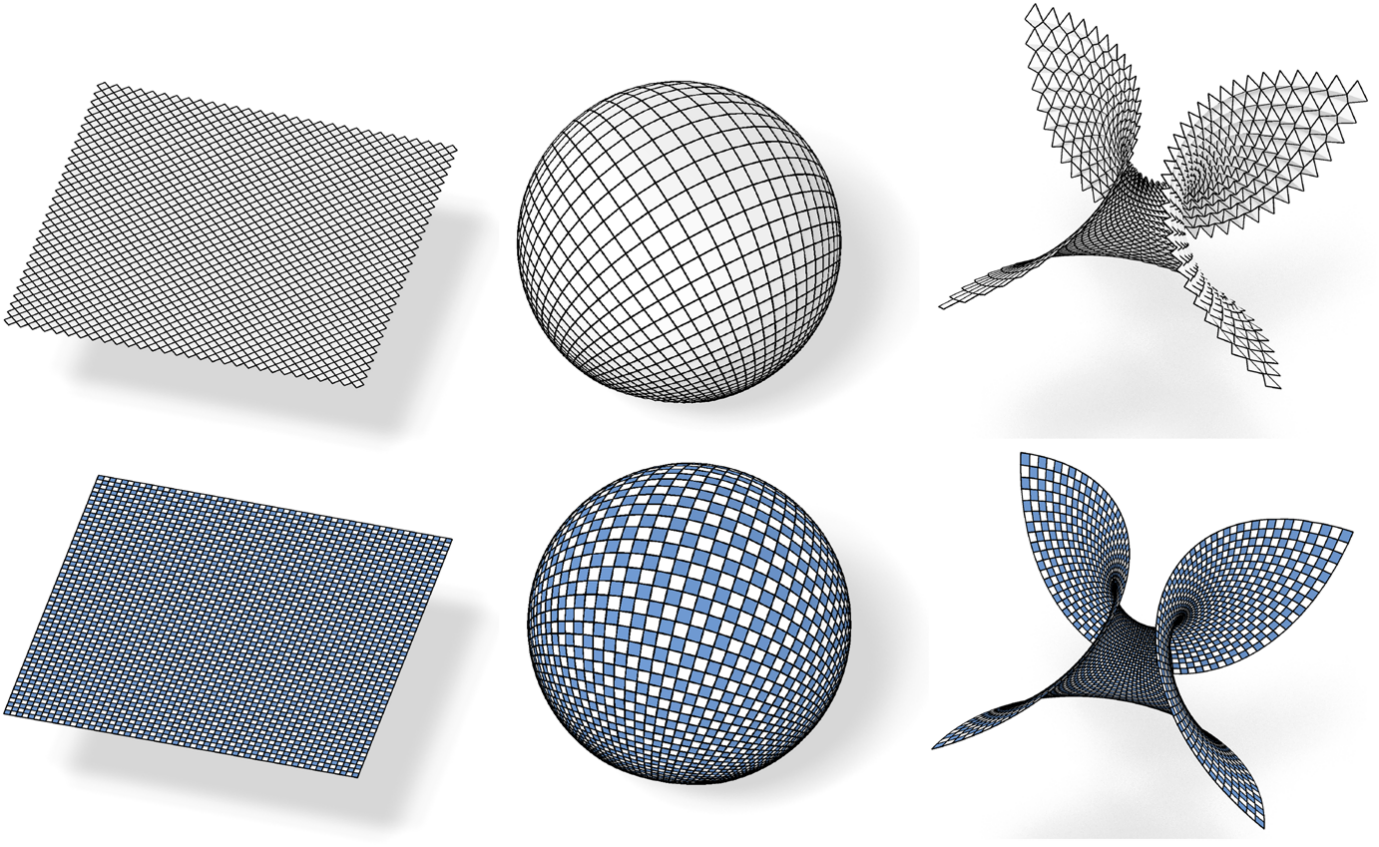
Enneper surface: In the top row we see from left to right the Weierstrass data of the Enneper surface, the Gauß image of the Ennepper surface and the Enneper surface itself. In the second row we see the checkerboard patterns of the corresponding nets

**Fig. 26:**
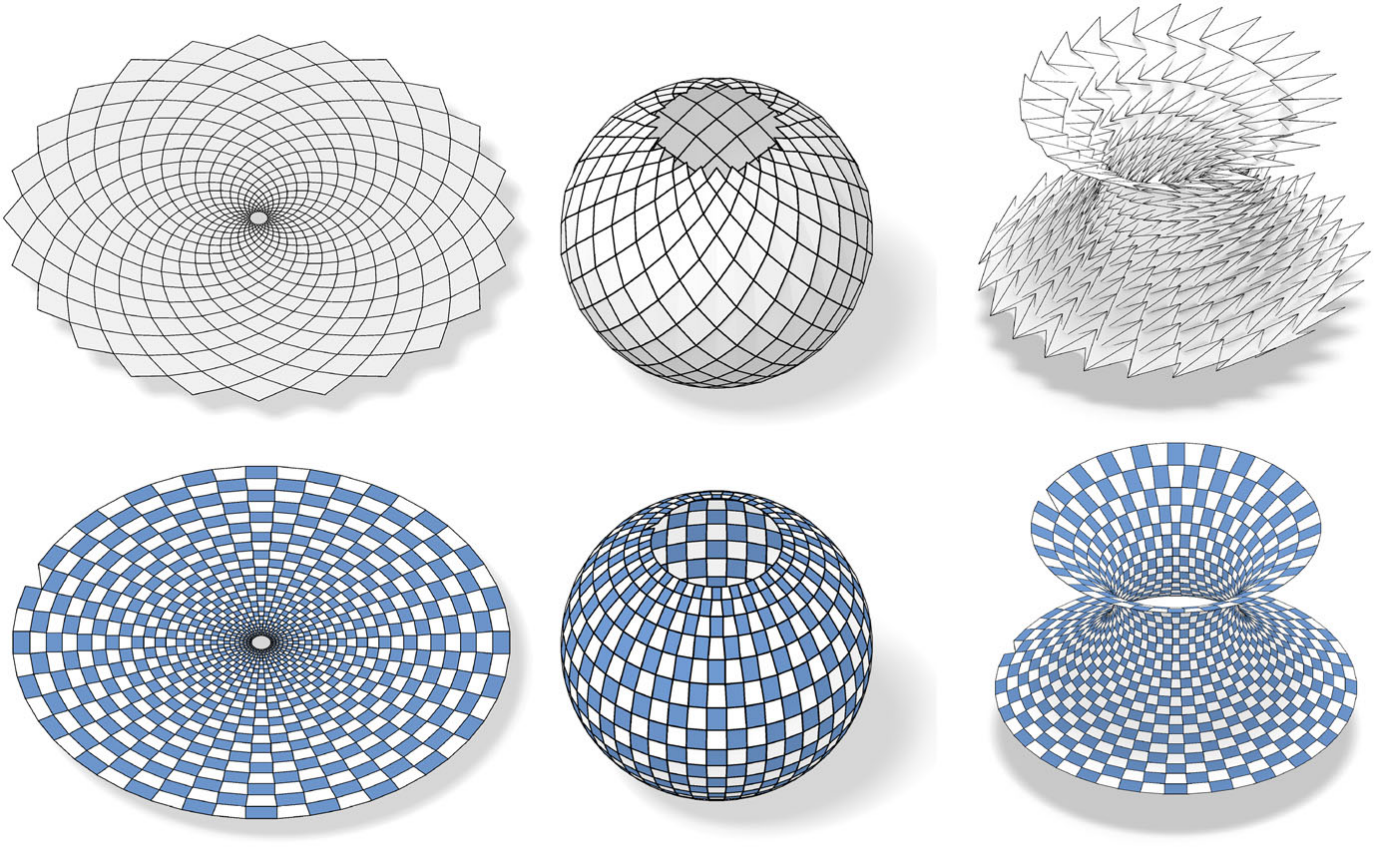
Catenoid: In the top row we see from left to right the Weierstrass data of the Catenoid, the Gauß image of the Catenoid and the Catenoid itself. In the second row we see the checkerboard patterns of the corresponding nets

**Fig. 27:**
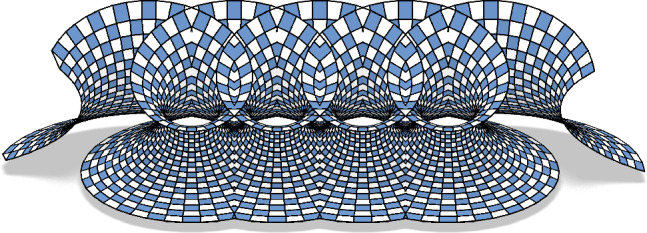
A Goursat transform of a periodically extended Catenoid

## Conclusion

In this paper we presented a novel discretization approach based on the checkerboard pattern inscribed to a quadrilateral net. On the one hand this allows a discrete curvature theory (Definition 3.3) that is compatible with discrete offsets (formula (3)) similar to [9, 15]. On the other hand this approach allows a new discretization of conjugate nets, orthogonal nets and principal nets (Definition 2.4). We showed several properties of these nets, most noticeably that principal nets are consistent with the curvature theory (Corollary 3.5) and are invariant under Möbius transformations (Theorem 4.4) applied to the corresponding sphere congruence introduced in [19].

Further the checkerboard pattern could be used to define discrete Koenigs nets using the conic of Koenigs (Definition 5.1) analogous to [7]. We find that discrete Koenigs nets are exactly those nets that are dualizable (Theorem 5.14) which links the approach taken in [7] to the approach of [5]. Other characterizations of discrete Koenigs nets that have been found in this paper are the existence of a closed multiplicative one-form defined on the edges of a checkerboard pattern (Theorem 5.5) similar to [5]. The characterization of Koenigs nets via the equality of Laplace invariants (Theorem 5.8) fits the original definition of these nets in the classical differential geometry. From the characterization via equal Laplace invariants we could deduce that the class of discrete Koenigs nets is invariant under projective transformations (Corollary 5.11).

Despite the discretization idea of Koenigs nets and principal nets being quite different they work well together for isothermic nets which are defined as principal Koenigs nets. This means that the Koenigs property is preserved upon Möbius transformations (Theorem 6.3) and the principality is preserved upon dualization (Corollary 6.2). Consequently we can apply Möbius transformations and dualizations to discrete isothermic nets. This allows a construction of discrete minimal surfaces from an isothermic net in the plane. First we map it to the unit sphere with a Möbius transformation, where it can be interpreted as the Gauß image of a minimal surface. Then it is dualized to gain the corresponding minimal surface from its Gauß image, compare both Figs. 25 and 26. If a Möbius transformation is applied to the Gauß image before it is dualized, we obtain a Goursat transform of the initial minimal surface, compare Fig. 27.

## Figures and Tables

**Fig. 28:**
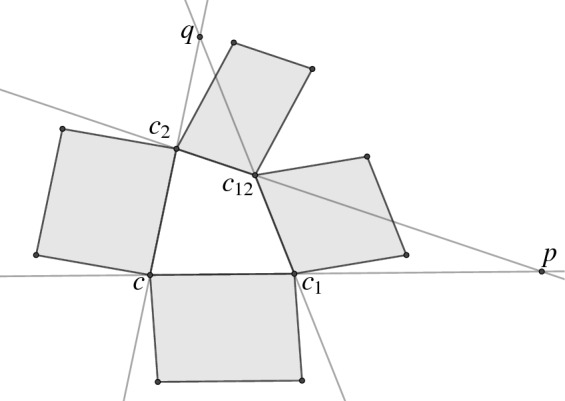
The multiplicative one form is automatically closed around every second order face as a consequence of Menelaus’ Theorem

**Fig. 29:**
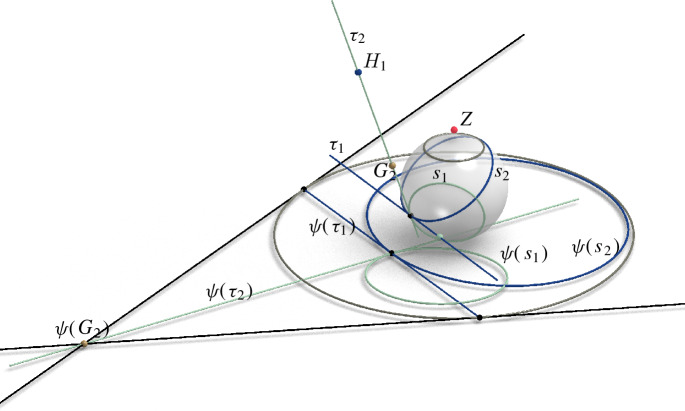
The two-dimensional case of Lemma B.1. The circles $s_1$ and $s_2$ on the unit sphere intersect orthogonally. The ellipses are their projections through the point *Z*. The gray ellipse $\psi ({\mathbb {S}}^2)^*$ is the contour of the unit sphere under the same projection. We see that the ellipses $\psi (s_1)$ and $\psi (s_2)$ intersect in conjugate lines with respect to $\psi ({\mathbb {S}}^2)^*$ as the polar point $\psi (G_2)$ of the tangent $\psi (\tau _2)$ is contained in $\psi (\tau _2)$. The preimage of this polar point drawn in beige is the intersection of the corresponding tangent line to the unit sphere with the polar plane of the center of projection. The gray circle on the unit sphere is the preimage of the contour quadric, i.e., the intersection of the unit sphere with the polar plane of *Z*

**Fig. 30:**
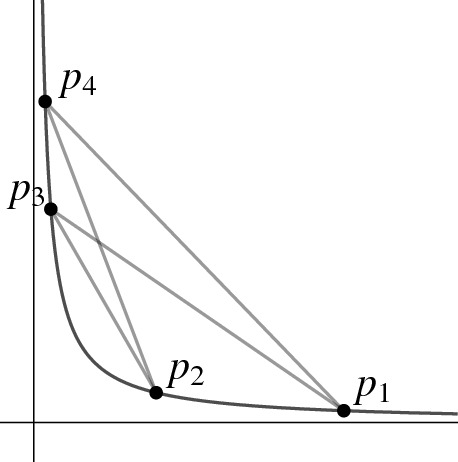
Inscribed angle theorem for hyperbolas. The four points $p_1$, $p_2$, $p_3$, and $p_4$ lie on a rectangular hyperbola if and only if the quotient of slopes of $\overline{p_4 p_2}$ and $\overline{p_4,p_1}$ equals the one of $\overline{p_3p_2}$ and $\overline{p_3p_1}$

## Data Availability

No specific data has been used for this article. The code written to produce the presented figures can be provided by the author on reasonable request.

## Appendix A: Proof of Theorem 5.5

### Proof

First we show that *q* is closed around every second order face, i.e., multiplying the contribution of every edge of a second order face in counter-clockwise order yields one. Let *c*, $c_1$, $c_{12}$, and $c_2$ be the vertices of a second order face, $p = \ell (c,c_1) \cap \ell (c_2,c_{12})$ and $q = \ell (c,c_2)\cap \ell (c_1,c_{12})$, see Fig. 28. Using Menelaus’ Theorem B.3 for the triangle $(c,c_1,q)$ and the triangle $(c_2,c_{12},q)$ we find that

Next we show that *q* is closed on the edges of every first order face $\mathcal {B}= (c,c_1,c_{12},c_2)$ if and only if the checkerboard pattern is a Koenigs net. As the multiplicative one-form *q* is projectively invariant, we choose a projective coordinate system such that $c = (0,0,1)$, $c_1 = (1,0,1)$, $c_2=(0,1,1)$, and $c_{12} = (1,1,1)$. The intersection points then have the following coordinates:  for suitable $s,t,v,u \in {\mathbb {R}}$. Those six points lie on a common conic section if the system of equations $Ax_i^2 + Bx_iy_i + Cy_i^2 + D x_iz_i + E y_iz_i + F z_i^2 = 0$ has a non-trivial solution. Here $x_i$, $y_i$, and $z_i$ stand for the three homogeneous coordinates of $p_i$. We compute the determinant of the matrix of this system of equations:

A non-trivial solution exists if and only if the determinant is zero. We can exclude the cases $t=0$ and $u=0$ since no $p_i$ are the same. We find that the determinant is zero if and only if

$$\begin{aligned}s - st + vst = v - vu + vus.\end{aligned}$$

Now we compute the multiplicative one-form along the edges of the quadrilateral. We find  and $p_1 = c_1 - c$. So if we use *c* and $c_1$ as the bases for the line $\ell (c,c_1)$, we obtain

For the next cross-ratio we express the points $p_2$ and $p_6$ via $c_1$ and $c_{12}$, obtaining $p_2 = -c_1 + c_{12}$ and . So the cross-ratio is

Next, equations $p_1 = c_{12} -c_2$ and  yield

and $p_2 = c_2 - c$ and  yield

Now *q* is closed if and only if

$$\begin{aligned}1= (1-t)\cdot \frac{v-1}{v}\cdot \frac{s}{s - 1}\cdot \frac{1}{1 - u}\iff s - st + stv = v - uv + vsu.\end{aligned}$$

Thus the existence of the conic of Koenigs is equivalent to *q* being closed. $\square$

## Appendix B: Some Theorems

The following lemma is known as trace polarity of a quadric. For a detailed description of trace polarity in German language see [6]. However, since a proof in English of the following lemma is hard to find, we give a proof in our setting (Figs. 29, 30).

### Lemma B.1

Let $s_1$ and $s_2$ be two orthogonally intersecting spheres on ${\mathbb {S}}^3$, i.e., intersections of ${\mathbb {S}}^3$ with conjugate hyperplanes $h_1$ and $h_2$. If $\psi$ is the central projection from point $Z\in {\mathbb {P}}{\mathbb {R}}^{4}$ onto a hyperplane $\zeta \cong {\mathbb {P}}{\mathbb {R}}^3$, then in this hyperplane $\psi (s_1)$ and $\psi (s_2)$ intersect in conjugate tangent planes with respect to the contour quadric $\psi ({\mathbb {S}}^3)^*$ of $\psi ({\mathbb {S}}^3)$. This means the corresponding tangent planes at the intersection points of $\psi (s_1)$ and $\psi (s_2)$ are orthogonal with respect to the inner product induced by $\psi ({\mathbb {S}}^3)^*$.

### Proof

For the proof we use homogeneous coordinates of ${\mathbb {P}}{\mathbb {R}}^4$. We choose a basis $(b_0,\dots , b_4)$ such that the center of projection $Z = b_0$. Let *Q* be the matrix such that the homogeneous coordinates of all points in ${\mathbb {S}}^3$ are given by $\{x\in {\mathbb {R}}^5 {\setminus } \{0\}: x^T Q x = 0\}$. We can assume without loss of generality that *Q* is a diagonal matrix. Since projections onto different planes are projectively equivalent, we can further assume that $\zeta$ is the polar hyperplane of *Z*. Thus $\zeta$ is given by the equation $x_0 = 0$. We introduce the block notation .

Since $\zeta$ is the polar hyperplane of *Z*, the contour quadric $\psi ({\mathbb {S}}^3)^*$ is the intersection of ${\mathbb {S}}^3$ with $\zeta$. In homogeneous coordinates it is given by $\{(0,\bar{x}) \in {\mathbb {R}}^5{\setminus }\{0\}: \bar{x}^T \overline{Q} \bar{x} = 0\}$.

Let $P \in s_1 \cap s_2$ be a point in the intersection of $s_1$ and $s_2$ and let $\tau$ be the tangent hyperplane to ${\mathbb {S}}^3$ in *P*. Then the tangent planes to $s_1$ and $s_2$ are given by $\tau _1=h_1\cap \tau$ and $\tau _2=h_2\cap \tau$. Note that two hyperplanes are conjugate with respect to a quadric, if and only if each contains the polar point of the other. Let $H_1\in h_2\cap \tau$ be the polar point of $h_1$ and let $H_2\in h_1\cap \tau$ be the polar point of $h_2$. The line $g_1:=\ell (P,H_2)$ lies in $\tau _1$ and intersects $\zeta$. We denote the intersection point by $G_1:=g_1\cap \zeta$. Analogously we define $G_2:=\ell (P,H_1)\cap \zeta$. The line $g:=\tau _1\cap \tau _2$ also intersects $\zeta$ and we denote the intersection point by *T*. Writing $A\vee B \vee C$ for the plane spanned by *A*, *B*, *C*, we have $\tau _1 = P \vee T \vee G_1$ and $\tau _2 = P \vee T \vee G_2$. Since $T, G_1, G_2\in \zeta$, we find that $\psi (\tau _1) = \psi (P) \vee T \vee G_1$ and $\psi (\tau _2) = \psi (P) \vee T \vee G_2$.

We now show the orthogonality of $\psi (\tau _1)$ and $\psi (\tau _2)$ with respect to $\overline{Q}$. In order to facilitate the notation we identify points with their projective coordinates. As the projection $\psi$ just sets the first coordinate of *P* to zero and due to the form of *Q*, we find that

$$\begin{aligned} G_1^T \overline{Q} \psi (P)&= G_1^T Q P = (\lambda _1 H_2 + \mu _1 P)^T Q P = 0,\\ G_2^T \overline{Q} \psi (P)&= G_2^T Q P = (\lambda _2 H_1 + \mu _2 P)^T Q P = 0,\\ G_1^T \overline{Q} T&= G_1^T Q T = (\lambda _1 H_2 + \mu _1 P)^T Q T = 0,\\ G_2^T \overline{Q} T&= G_2^T Q T = (\lambda _2 H_1 + \mu _2 P)^T Q T = 0,\\ G_1^T \overline{Q} G_2&= G_1 Q G_2 = 0. \end{aligned}$$

Hence, the point $G_1$ is the polar point of $\psi (\tau _2)$ with respect to $\psi ({\mathbb {S}}^3)^*$ in $\zeta$ and vice versa. This shows the conjugacy of the tangent planes $\psi (\tau _1)$ and $\psi (\tau _2)$ of the projected spheres $s_1$ and $s_2$. $\square$

### Theorem B.2

(inscribed angle theorem for hyperbolas)  Consider ${\mathbb {R}}^2$ and coordinates (*x*, *y*) with respect to a basis. The slope of a vector (*a*, *b*) is defined as *b*/*a* for $a\ne 0$. Four points $p_i= (x_i,y_i) \in {\mathbb {R}}^2$ with $x_j \ne x_k$ and $y_j \ne y_k$ lie on a hyperbola with equation $y = {c}/{x}$ if and only if the quotient of slopes of $\ell (p_4, p_2)$ and $\ell (p_4,p_1)$ equals the quotient of slopes of $\ell (p_3,p_2)$ and $\ell (p_3, p_1)$, compare Fig. 30. Computing this condition yields

B1

### Theorem B.3

(Menelaus’ Theorem)  Let *A*, *B*, and *C* be the vertices of a triangle and let *g* be a straight line. For the three vertices $D = \ell (A,B) \cap g$, $E = \ell (B,C) \cap g$, and $F = \ell (C,A) \cap g$ the equation

$$\begin{aligned} \frac{(D-A)}{(B-D)} \cdot \frac{(E-B)}{(C - E)} \cdot \frac{(F-C)}{A - F} = -1 \end{aligned}$$

holds.
